# Seedless 3′ pairing enables miR-17 family miRNAs to seize let-7 target sites

**DOI:** 10.1093/nar/gkag767

**Published:** 2026-08-14

**Authors:** Artur Laski, Anneke Brümmer, Matije Lucic, Elisa Federici, Iris Mestres-Pascual, Jochen Imig, Alexander Kanitz, Mihaela Zavolan, Luca F R Gebert, Ian J MacRae, Carlo V Catapano, Jonathan Hall

**Affiliations:** Institute of Pharmaceutical Sciences, Department of Chemistry and Applied Biosciences, ETH Zurich, Zurich, 8093, Switzerland; Bioinformatics Competence Centre, University of Lausanne, Lausanne, 1015, Switzerland; Institute of Pharmaceutical Sciences, Department of Chemistry and Applied Biosciences, ETH Zurich, Zurich, 8093, Switzerland; Tumor Biology and Experimental Therapeutics, Institute of Oncology Research, Università della Svizzera Italiana, Bellinzona, 6500, Switzerland; Biozentrum, University of Basel, Spitalstrasse 41, Basel 4056, Switzerland; Max Planck Institute of Molecular Physiology, Chemical Genomics Centre, Dortmund 44227, Germany; Biozentrum, University of Basel, Spitalstrasse 41, Basel 4056, Switzerland; Biozentrum, University of Basel, Spitalstrasse 41, Basel 4056, Switzerland; Department for Integrative Structural and Computational Biology, The Scripps Research Institute, La Jolla, CA 92037, United States; Department for Integrative Structural and Computational Biology, The Scripps Research Institute, La Jolla, CA 92037, United States; Tumor Biology and Experimental Therapeutics, Institute of Oncology Research, Università della Svizzera Italiana, Bellinzona, 6500, Switzerland; Institute of Pharmaceutical Sciences, Department of Chemistry and Applied Biosciences, ETH Zurich, Zurich, 8093, Switzerland

## Abstract

MicroRNAs (miRNAs) are short noncoding RNAs that post–transcriptionally regulate gene expression. Their canonical function depends on a “seed” sequence at the miRNA 5′ end that pairs with conserved sites in the 3′ untranslated regions of target messenger RNAs to suppress gene expression. Although seed–mediated targeting is well characterized, the role of the miRNA 3′ end remains poorly understood and is challenging to study. Here, we identify a noncanonical mechanism by which miR–106a, a member of the miR–17 family, competes with let–7a for binding to let–7 target sites despite their distinct seed sequences. This binding bypasses miR–106a seed pairing and instead relies on extensive complementarity at its 3′ end, which shares sequence identity with the let–7a seed. Such binding of miR–106a does not itself silence these targets but shields them from let–7–mediated suppression, resulting in de-repression of let–7 targets. By contrast, miR–106b, which differs from miR–106a and the other miR–17 family members at its 3′ end, lacks this de-repressive activity. These findings show that variation in miRNA 3′ sequences can generate divergent functions within a miRNA family and underscore the need to account for 3′–end contributions when interpreting miRNA–target networks and considering the development of miRNA–based therapeutics.

## Graphical Abstract

**Graphical Abstract:**
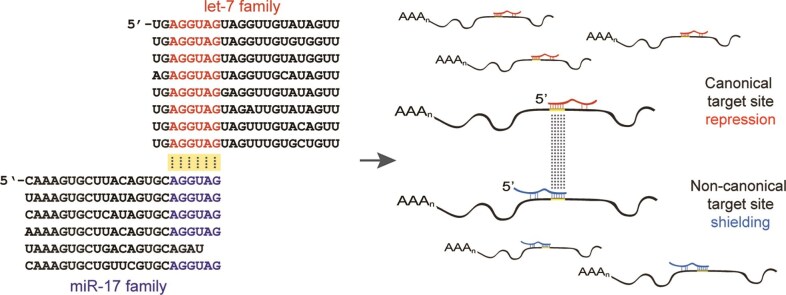

## Introduction

MicroRNAs (miRNAs) are small noncoding RNAs that regulate gene expression post-transcriptionally by guiding Argonaute (Ago) proteins to partially complementary sequences within messenger RNAs (mRNAs) [1]. In animals, target recognition typically depends on base pairing between nucleotides 2–8 of the miRNA, the so-called seed region [2, 3], and complementary sites located in the 3′ untranslated region (3′-UTR) of the mRNA. The seed region is preorganized within Ago for target binding [4, 5], and pairing to this region alone is often sufficient to mediate repression. Canonical target sites are classified based on their seed match architecture: 8mer sites, which pair to nucleotides 2–8 of the miRNA and include an adenosine opposite position 1, exhibit the strongest repression, followed by 7mer and 6mer sites [6, 7].

**Figure 1.**
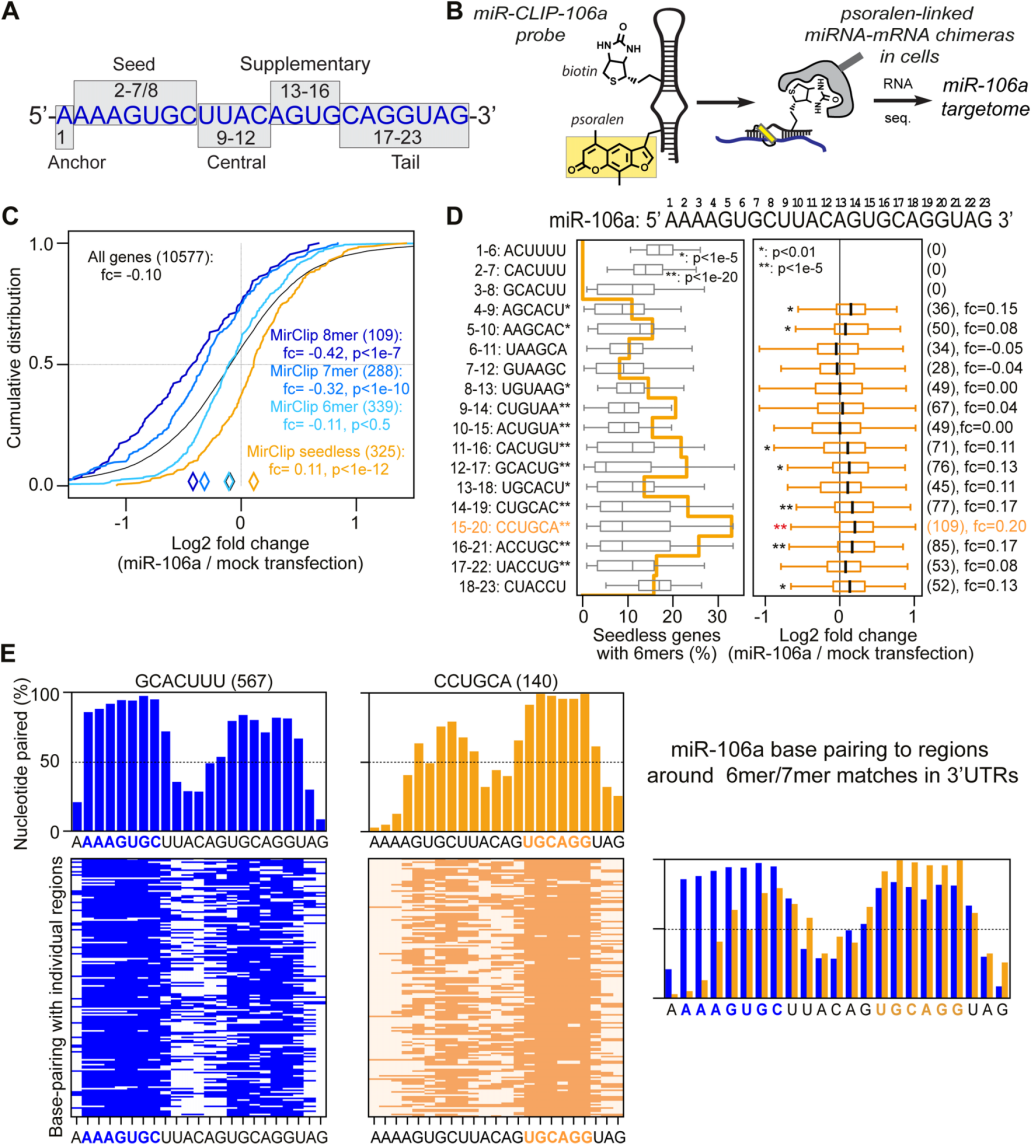
miR-CLIP-106a captured mRNAs enriched for 6mers complementary to the 3′ end of miR-106a that increase upon miR-106a transfection. (**A**) Functional domains of miRNA, exemplified by a numbered sequence for miR-106a. (**B**) Schematic of the miR-CLIP method [33] performed on miR-106a. miRNA probes bearing psoralen and biotin are transfected into HeLa cells. After 24 h, Ago2 immunoprecipitation is performed, followed by streptavidin affinity purification to isolate probe-linked RNAs. The RNA is then converted into complementary DNA and sequenced to reveal both conventional and nonconventional miRNA targets. (**C**) Cumulative distributions of log_2_ fold changes in transcript abundances following miR-106a transfection. The fold changes of all transcripts (black) are compared to four groups within the top 1500 miR-CLIP targets: targets with 8mer seed match (dark blue), 7mer seed match (medium blue), 6mer seed match (light blue), and “seedless targets” (orange, no seed matches to nucleotides 1–6, 2–7, or 3–8 in the 3′ UTR). Leftward shifts indicate stronger target downregulation. Diamonds mark the median fold change for each group. *P-*values are from Wilcoxon rank-sum test. (**D**) Left panel: Orange line: percentage of seedless mRNAs [orange group in (Fig. 1C)] with reverse-complement 6mer matches to the miR-106a sequence, indicated 6mers on the *y*-axis, in their 3′ UTRs, Gray boxes: percentages of seedless mRNAs with matches to shuffled 6mers with the same nucleotide composition. *P-*values were calculated using a two-sided one-sample *t*-test to compare the percentage of mRNAs with a 6mer match (orange line) to those with matches to shuffled 6mers (gray boxes). Right panel: Boxplots of log_2_ fold changes after miR-106a transfection for mRNAs with 6mer matches to the miR-106a sequence. *P-*values are calculated using a two-sided Wilcoxon rank-sum test to compare fold changes with those of all transcripts [black curve in panel (C)]. 6mer matches complementary to nucleotides 15–20 of miR-106a (orange font) were most frequent (109 out of 325 mRNAs, 34%), and these mRNAs also showed the strongest upregulation. (**E**) Predicted base-pairing between miR-106a and selected target regions using RNAhybrid, allowing bulges of 1 nt and internal loops of up to 15 nt [46]. Left panels: Predicted base-pairing of miR-106a to 567 mRNA regions (47 nt in length) centred around GCACUUU seed matches in 3′ UTRs of transcripts suppressed by miR-106a in HeLa cells [dark and medium blue groups in (Fig. 1C)]. Middle panels: Predicted base-pairing of miR-106a to 140 sequences (46 nt in length) from 109 seedless target transcripts containing CCUGCA complementary to U15–G20 in miR-106a. Top graphs show the frequency of pairing for each nucleotide in the miR-106a sequence, and bottom panels show paired bases of miR-106a (*x*-axis) for each target region (in random order, *y*-axis). Base-pairing interactions were from the minimum free energy (MFE) miRNA–RNA hybrid structures determined using RNAhybrid, allowing bulges of 1 nt and loops of up to 15 nt. Right panel: Direct comparison of base-pairing frequencies at each miR-106a nucleotide between 7mer seed-match target sites (blue) and seedless target sites (orange).

Ago binds the 5′ end of the miRNA in its Middle domain and anchors the 3′ end in the Piwi/Argonaute/Zwille (PAZ) domain [8, 9] (Fig. 1A). This Ago–miRNA complex, together with effector proteins such as GW182/TNRC6, forms the RNA-induced silencing complex (RISC) [10, 11]. RISC facilitates translational repression or mRNA decay by recruiting the CCR4–NOT deadenylation complex and the poly(A)-binding protein [12, 13]. Although the seed-centric model accounts for a substantial portion of miRNA activity, high-throughput crosslinking and immunoprecipitation (CLIP) studies have consistently identified Ago–miRNA-mRNA interactions that lack contiguous seed pairing [14–19]. These noncanonical interactions are often stabilized by pairing to the 3′ region of the miRNA and occur in a context-specific manner. Despite being frequently observed in Ago CLIP datasets, such interactions often fail to produce measurable repression at the mRNA level in bulk assays [6]. However, structural and biochemical studies suggest that 3′-end pairing can stabilize otherwise transient interactions and may enable functional regulation in specific cases [7, 20–22].

This alternative targeting mode is particularly relevant for understanding specificity within miRNA families. Approximately 60% of human miRNAs belong to families whose members share identical seed sequences, resulting in substantial functional redundancy [23]. However, family members often diverge in their 3′ regions, and in some cases, pairing outside the seed can discriminate among otherwise indistinguishable miRNAs. For example, in C. *elegans*, let-7 paralogs differentially regulate lin-41 through distinct 3′ pairing architectures [24]. Strand selection and isoform diversity further expand targeting potential, as iso-miRs generated by alternative Drosha or Dicer processing diversify the target repertoire of a single locus [25].

These observations challenge key assumptions underlying most predictive models. Although supported by CLIP and biochemical interaction data [26], such models tend to prioritize canonical seed complementarity and evolutionary conservation [6, 27], thereby potentially underrepresenting noncanonical interactions such as tail-mediated or seedless binding and highlighting the need for more refined approaches.

In recent years, specialized Ago-CLIP methods have provided direct experimental access to miRNA–mRNA interactions in cells, enabling the systematic detection of noncanonical binding events [28]. These data reveal that miRNA target recognition extends beyond canonical seed pairing and give rise to an expanded and previously underappreciated miRNA targetome. Importantly, such expansion increases the likelihood of overlapping and competitive interactions between miRNAs, particularly between family members that share related targeting space. At the biological level, this complexity is reflected in the opposing functions of major miRNA families in development and human disease. The miR-17∼92 cluster and its paralogs miR-106∼363 and miR-106b∼25 encode several oncogenic miRNAs with overlapping seeds [29, 30]. In contrast, the broadly expressed and evolutionarily conserved let-7 family, comprising nine members, acts as a tumor suppressor by targeting oncogenes such as HMGA2, IGF2BP1, and LIN28B [31, 32]. These two prominent miRNA families therefore drive major and functionally opposing regulatory programs in mammalian cells, yet whether and how their interactions intersect at the level of individual target sites remains unclear.

To interrogate noncanonical interactions at transcriptome scale, we developed miR-CLIP, a photo-crosslinking approach that captures miRNA target transcripts in cells (Fig. 1B) [33]. In miR-CLIP, a pre-miRNA that is functionalized with psoralen- and biotin groups is transfected at low concentrations into cells, is processed by RISC, and is activated by brief mild photo-irradiation for target capture. MiRNA–mRNA chimeras are subsequently enriched by immunoprecipitation and streptavidin capture prior to RNA sequencing. MiR-CLIP has been used to identify the canonical targetomes of miR-106a in HeLa cells [33], miR-124, iso-miR-124, and miR-132 in HEK 293T cells [25], and miR-29a in keratinocytes [34]. Furthermore, miR-CLIP revealed that the long non-coding RNA H19 is an unconventional target of miR-106a [33], and that sub-targetomes of miR-124 and iso-miR-124 are functionally distinguished by a bulged guanosine in the seed-binding target region of miR-124 [25]. Neither of these findings could have been readily predicted using currently available *in silico* methods, which are primarily designed to account for the majority of observed interaction data and will miss noncanonical or context-dependent events.

In the original miR-CLIP study [33], we discovered a set of targets captured by miR-CLIP-106a that did not carry a complementary sequence to the miR-106a seed. In this present study, we explain how these findings derive from a distinct mode of target recognition that relies on extensive 3′ pairing in the absence of canonical seed complementarity. Using Ago CLIP, transcriptomic profiling, and cellular assays, we show that miR-106a binds and regulates a population of seedless targets stabilized by pairing to its tail region. Many of these sites are also recognized by let-7a, and we demonstrate that miR-106a can limit let-7a engagement with these targets, suggesting a novel mode of functional antagonism. These findings reveal a cryptic, tail-driven targetome and demonstrate that sequences outside the seed can govern specificity within miRNA families.

## Materials and methods

### Cell culture

HEK 293T cells (ATCC, #CRL-3216) were cultured as adherent monolayers in Dulbecco’s Modified Eagle’s Medium (Gibco, #31966021) supplemented with 10% fetal bovine serum (FBS; Gibco, #10270106). Cells were maintained at 37°C in a humidified atmosphere containing 5% CO_2_. DU145 prostate cancer cells were maintained in RPMI 1640 medium (21875-034, Gibco®) supplemented with L-Glutamine 2%, 10% FBS (S1810-500, Biowest), and 1% penicillin–streptomycin (15140-122, Gibco®). Cells were regularly checked for *Mycoplasma* contamination using MycoAlert *Mycoplasma* detection kit (Lonza).

### Oligonucleotide synthesis and purification

All RNA hairpins, RNA duplexes, siRNAs, and DNA oligonucleotides were synthesized and purified as previously described [35]. Synthetic RNA mimics were purchased from Dharmacon (Horizon Discovery) as HPLC–purified, desalted products, and delivered in lyophilized form prior to reconstitution in nuclease–free water. miRNA inhibitors (miRVana, Thermo Fisher Scientific) were obtained as chemically modified antisense oligonucleotides and reconstituted in nuclease-free water. The sequences of the synthesized oligonucleotides are listed in Supplementary Table S1.

### HEK293T cell seeding and transfection

HEK 293T cells were seeded 6–8 h prior to transfection in either, six-well plates (350 000 cells/well for total RNA isolation following antimir treatment), 12-well plates (150 000 cells/well; for quantitative polymerase chain reaction (qPCR), western blot, or transcriptomic analysis), or 96-well opaque plates (10 000 cells/well; for luciferase assays). Transfections were performed using Lipofectamine 2000 (Invitrogen, #11668027) with final oligonucleotide concentrations up to 50 nM, following the manufacturer’s instructions. Complexes were formed by incubating reagents for ∼25 min before adding to cells. Incubation proceeded for up to 48 h.

### RNA isolation and RT-qPCR

HEK 293T cells were washed with ice-cold phosphate buffered saline and lysed in TRIzol reagent (Invitrogen, #15596-018). RNA was extracted per the manufacturer’s protocol and reverse-transcribed using the TaqMan MicroRNA RT Kit (Thermo Fisher Scientific, #4366596) with a 1:1 mix of random hexamer (Promega, #C1181) and oligo (dT)15 primers (Promega, #C1101). qPCR was performed using KAPA SYBR FAST qPCR master mix (Kapa Biosystems, #KK4618) on a LightCycler 480 system (Roche). Each sample was run in technical triplicates. Relative expression levels were calculated using the 2^–ΔΔCp method, with ACTB and GAPDH serving as reference genes. The sequences of qPCR primers are provided in Supplementary Table S2.

### Western blotting

HEK293T cells were lysed in RIPA buffer (Sigma–Aldrich, #R0278), and protein concentration was determined using the BCA Protein Assay Kit (Thermo Fisher Scientific, #23227). Equal amounts of protein (20–30 µg) were resolved by sodium dodecyl sulfate–polyacrylamide gel electrophoresis and transferred to PVDF membranes (Thermo Fisher Scientific, #88518). Membranes were blocked in 5% milk and probed overnight at 4°C with primary antibodies against LIN28B (1:1000) or GAPDH (1:10 000). After washing, HRP-conjugated secondary antibodies were applied for 2 h at room temperature. Signal was developed using the Amersham ECL detection reagent (GE Healthcare, #RPN2109) and imaged with a CCD camera (Bio-Rad). The list of antibodies used is provided in Supplementary Table S4.

### Luciferase assays

HEK293T cells were plated in opaque 96-well plates and transfected with specified RNA concentrations using Lipofectamine 2000 (Invitrogen, #11668027), following the manufacturer’s guidelines. Transfections were conducted in sets of technical triplicates. Twenty-four hours after the RNA transfection, 20 ng/well of the reporter plasmid was introduced into the cells using JetPEI (Polyplus, #101-10), according to the manufacturer’s instructions. Forty-eight hourspost-plasmid transfection, cells were lysed using the Dual-Glo® Luciferase Assay System (Promega, #E2940), with the following adjustments to the standard protocol: the Dual-Glo® Luciferase Reagent was diluted 1:1 with water and used at 30 μl per well, while the Dual-Glo® Stop & Glo® Reagent was applied at a volume of 15 μl per well. Luminescence readings were taken with a Mithras LB940 plate reader (Berthold Technologies, Bad Wildbad, DE). Normalization of the data was done relative to the firefly luciferase activity and the 0 nM RNA control.

### Reporter construct cloning and transfection

The psiCHECK-2 dual-luciferase plasmid (Promega, #C8021) was linearized with NotI and XhoI (Promega, #R6431 and #R6161) and ligated with inserts targeting the 3′UTR of *Renilla* luciferase. Constructs were verified by sequencing. The sequences of the cloned inserts are listed in Supplementary Table S3.

### Live-cell analysis of cell proliferation assay

DU145 cells were plated in 96-well plates at a density of 900 cells/well. Cells were transfected 24 h later with miRNAs. The final concentration of targeted miRNA was 25 nM. For co-transfections, 25 nM of each miRNA was used, with a total miRNA concentration of 50 nM. To maintain the total concentration of transfected miRNA constant, in the single-transfection conditions, a negative control miRNA (miCon) was co-transfected at 25 nM with the targeted miRNA to maintain the total miRNA concentration of 50 nM.

miRNAs were diluted in Opti-MEM (Gibco) and transfected into cells using INTERFERin (Polyplus) following the manufacturer’s protocol. Cells were incubated for 6 days and monitored using the IncuCyte® Live-Cell Analysis System (Sartorius). Images were collected using a 10× objective. Mean confluence was calculated from four technical replicates by the IncuCyte software using four non-overlapping bright phase images for each replicate. Data are presented as mean ± SD. To assess statistically significant differences between groups, two-way analysis of variance (ANOVA) analysis was performed using GraphPad Prism software (v10.2.3).

### RNA integrity and quantification

Total RNA concentration was measured using the QuantiFluor RNA System (Promega, #E3310), and integrity was assessed on the Bioanalyzer (Agilent) using the RNA 6000 Nano Chip (Agilent, #5067-1511). RNA Integrity Number values averaged 8.8.

### RNA-Seq library preparation and sequencing of miRNA-transfected samples

Libraries were prepared from 200 ng of total RNA using the TruSeq Stranded mRNA Library Prep Kit (Illumina, #RS-122-2103). Library quality was confirmed using the Fragment Analyzer (Advanced Analytical) and the Standard Sensitivity NGS Fragment Kit (#DNF-473). Libraries were pooled to equal molarity and quantified using the QuantiFluor ONE Double-Stranded DNA System (Promega, #E4871). Final pools were diluted to 1.4 pM and sequenced on a NextSeq 500 (Illumina) using the High Output 75-cycle kit (#FC-4041005) in single-end 76-bp format with dual 8-bp indexes.

### RNA-Seq library preparation and sequencing of anti-miR-transfected samples

For total RNA isolation, frozen cell pellet was dissolved in 1 ml TRI Reagent® (Sigma: T9424-200) and incubated for 5 min at room temperature. Thereafter, RNA was isolated using the Direct-zol RNA MiniPrep kit (Cat. No. R2050, Zymo Research) according to the manufacturer’s protocol, including DNase treatment on column. RNA was eluted in 50 µl of elution buffer and submitted to the Genomic facility DBSSE, Basel, for library preparation and sequencing. The quantity of RNA was measured with RNA BR Qubit Kit (Invitrogen), and their integrity was monitored on TapeStation RNA SS kit (Agilent). Two hundred nanograms total RNA was used for library preparation using TruSeq Stranded mRNA Library Prep kit (Illumina, Cat# 20020595) according to kit manual, where RNA fragmentation was done for 8 min at 94°C. Final PCR was done for 15 cycles. After library preparation, samples were pooled to equal molarity. The pool was sequenced single-end 76 bases (in addition: 8 bases for index 1 and 8 bases for index 2) on NextSeq 500 using the NextSeq 500 High Output Kit 75-cycles (Illumina, Cat# FC-404-1005).

### mRNA expression quantification

RNA-Seq reads were aligned to the GRCh38 genome with transcript annotations (both downloaded from https://www.gencodegenes.org/human/) using STAR (v2.7.10b) [36]. More than 88% of reads aligned uniquely per sample on average.

RNA-Seq reads were counted per gene using HTSeq (v2.0.9.) [37] with intersection-strict mode and ignoring non-unique alignments. Gene read counts were normalized to one million for each sample. Gene expression fold changes between miRNA transfection conditions were calculated for each gene after averaging the log_2_ normalized reads counts over three replicates per condition. Gene expression fold changes between anti-miRNA and mock transfection were calculated with DESeq2 [38].

### miRNA-Seq quantification

Genome and transcriptome sequences of *Homo sapiens* (GRCh38.p14, Ensembl release 111) were obtained from Ensembl (https://ensembl.org). Only transcripts supported by Ensembl transcript support level (TSL) 1–3 were considered. Canonical precursor and mature miRNA annotations were retrieved from miRBase (v22) (https://mirbase.org/) [39]. To enable detection of mutant miRNAs, the sequences of transfected miRNA precursors were appended to the genome and to the gene and miRNA annotation files.

Small RNA sequencing data were processed using MIRFLOWZ (v0.9.0). Adapter sequences (AACTGTAGGCACCATCAA) were trimmed, and reads ≥ 15 nt were retained. Then, reads were aligned to both genome and transcriptome references using two aligners: segemehl (v0.3) [40] and Oligomap (v1) [41].). All alignments were merged and filtered to retain only alignments with the minimal edit distance per read. Reads mapping to >100 loci were excluded.

To account for the higher frequency of insertions and deletions (InDels) in miRNA loci [42–44], multimapping reads (i.e. those aligning with equal edit distance to multiple locations) and alignments were retained only if the number of InDels exceeded or equaled the number of mismatches. Filtered alignments were intersected with miRNA annotations using BEDTools (v2.30) [45].

To accommodate isomiR length variability, we allowed a 3-nt tolerance at both ends of the alignments relative to annotated precursor and mature miRNAs. Each alignment was weighted by 1/N, where N is the number of genomic loci that the read mapped to with equal score and InDel characteristics. This ensured that no individual read contributed more than one count in total.

Counts were normalized to library size (counts per million) for each sample and averaged across three replicates. For visualization, the 15 most abundant sequences per locus were shown.

### Analysis of miR-CLIP data for miR-106a obtained in HeLa cells from Imig *et al*. (2015)

Abundance levels of transcripts in miR-106a miR-CLIP samples and upon pre-miR-106a transfection in HeLa cells were downloaded from the Gene Expression Omnibus (GEO; accession codes: GSE62675 and GSE62678, respectively). Log_2_ fold changes after miR-106a compared to control transfection were calculated for all transcripts. The top 1500 transcripts with highest abundance levels in the final miR-106a miR-CLIP sample were grouped into those with canonical seed matches to miR-106a in their 3′ UTR sequence: 8mer targets with GCACUUUA, 7mer targets with GCACUUU, and 6mer targets with CACUUU; and those without a 6mer match to the miR-106a seed region (neither CACUUU, ACUUUU, nor GCACUU) in their 3′ UTR were grouped together as “Seedless” transcripts.

Seedless transcripts were counted with reverse complementary 6mer matches to the miR-106a sequence beyond the seed region in their 3′UTR sequence. As random control, for each 6mer from the miR-106a sequence, we also counted the reverse complementary matches to shuffled 6mers in the 3′ UTRs (excluding shuffled 6mers that were identical to 6mers from the miR-106a sequence). Similarly, we counted reverse complementary matches to 6mers from the let-7a sequence and to shuffled 6mers in the 3′ UTRs of Seedless transcripts, excluding shuffled 6mers that were found elsewhere in the let-7a or miR-106a sequences.

### Predicted base-pairing between miRNA and mRNA target sequences using RNAhybrid (2006)

MFE miRNA–mRNA hybrid structures were predicted using RNAhybrid [46] with the following options: -b 1 -v 1 -c -s 3utr_human, i.e. allowing bulges of 1 nt. By default, RNAhybrid allows internal loops of up to 15 nt in target and miRNA sequences, G-U-wobble base-pairing, and imposes no helix constraints (i.e. no prioritizing of miRNA seed interactions over other base interactions in the MFE computation). As canonical target sequences we considered 47-nt-long sequences centered around 7mer seed matches (GCACUUU) in the 3′ UTRs of the top 1500 miR-106a miR-CLIP-captured transcripts. As seedless, noncanonical, target sequences, we considered 46-nt-long sequences centered around the most frequent 6mer (CCUGCA) from the miR-106a sequence found in the 3′ UTRs of 109 Seedless transcripts. Base-pairing was predicted for miR-106a, let-7a, their family members, and for 40 miRNAs with highest abundance in HeLa cells as quantified by Beuvink *et al*. [47].

### Analysis of chimeric eCLIP data obtained in native HEK 293T cells by Manakov *et al*. (2022)

Chimeric eCLIP data (peaks, genome browser tracks, and raw fastq files) generated by Manakov *et al*. [48] in HEK 293T cells were downloaded for miR-17 and let-7a from GEO (accession codes: GSM5942126 and GSM5942101, respectively). Note, that eCLIP data for miR-106a were not generated due to the low expression level of miR-106a in HEK 293T cells.

In Figs 5 and 7E–I, eCLIP targets for miR-17 and let-7a were defined based on a chimeric eCLIP peak (*P*-value < .00001, calculated by Manakov *et al*.) in their 3′ UTR that contained a 7mer seed match (GCACUUU for miR-17 and CUACCUC for let-7a) within the 40 nt around the center of the eCLIP peak. Only eCLIP targets unique to either miR-17 or let-7a were considered.

For the list of candidates of the miR-106a-let-7a interactome in HEK 293T cells (Supplementary Table S5 and Fig. 6A and B), genes with eCLIP reads were those with a chimeric eCLIP peak (*P* < .01) in their 3′ UTR with no further requirement for a seed match.

### Analysis of RNA-Seq and small RNA-Seq data obtained after miRNA transfection in HEK 293T cells

FASTQ files for three replicates each of 8 miRNA single transfections, 6 miRNA co-transfections, and 2 control transfections were aligned to the human genome (assembly hg38) using STAR [36]. Reads aligning to mRNAs were counted using htseq [37]. TPM (transcripts per million) levels of mRNAs were calculated by normalizing read counts to the mRNA length and then to the sum of length-normalized read counts per sample, and multiplying by 1 million.

Log_2_ fold changes after miRNA transfection compared to negative control transfection were calculated with the average TPM levels across the three replicates.

TargetScan target predictions [49] were downloaded from https://www.targetscan.org/vert_80/vert_80_data_download/Predicted_Targets_Context_Scores.default_predictions.txt.zip. Predicted targets were defined based on a weighted context score percentile over 75% for either miR-106a or let-7a. Predicted targets of both miRNAs were excluded.

### AlphaFold prediction

AlphaFold3 [50] was used through the AlphaFold server (alphafoldserver.com), with human Ago2 (Uniprot: Q9UKV8), miR-106a (5′-AAAAGUGCUUACAGUGCAGGUAG), and a putative binding site from HMGA2 (5′-AAAAUACCUCAAAUUAAGCAUAUGUGUU) as inputs. Five predictions were generated, and one each with C17 flipped in or flipped out, respectively, was depicted. Images were made in ChimeraX.

### Preparation of recombinant Ago2 loaded with specific guide RNAs

Human Ago2 was expressed in Sf9 cells according to a previously published protocol using the baculovirus system [51]. Cells were pelleted, resuspended in lysis buffer (50 mM NaH2PO4, pH 8, 100 mM NaCl, and 0.5 mM TCEP), and lysed by three passages through an M-110P microfluidizer (Microfluidics) cooled with ice. The lysate was clarified by centrifugation at 20 000 × *g* for 20 min, and used for a first purification by Ni affinity (Ni NTA agarose, Qiagen), with an on-resin RNase digestion using Micrococcal nuclease (Takara). After elution with 300 mM imidazole, Ago2 was loaded with single-stranded 5ʹ phosphorylated RNA guides, and the His tag was removed using Tobacco Etch Virus protease (prepared in the lab). Ago2 loaded with a specific guide was captured using an antisense oligonucleotide (IDT) and eluted with DNA competitor (IDT). Excess competitor was removed with Q Sepharose Fast Flow resin (Cytiva) and buffer-exchanged into storage buffer (50 mM Tris–HCl, pH 8, 100 mM NaCl, 0.5 mM TCEP). Concentration was determined on Nanodrop OneC (Thermo Fisher Scientific) using calculated extinction coefficients. Aliquots were flash-frozen in liquid nitrogen and stored at −80°C until use.

### T7 *in vitro* transcription and radiolabeling

DNA templates were ordered from IDT, annealed, and T7 prepared in the lab was used for an overnight reaction at 37°C. The transcription reactions were cleaned with a Monarch kit (NEB), followed by treatment with DNase I (NEB). A second purification by Monarch kit was followed by treatment with CIP to remove the 5ʹ triphosphate. This was followed by a final purification by Monarch kit.

The RNAs were radiolabeled using gamma 32P ATP (Revvity) and T4 PNK (NEB), and purified by urea polyacrylamide electrophoresis. Finally the labeled RNAs were quantified by Nanodrop OneC (Thermo Fisher Scientific).

### Dot blot binding assays

Reactions were prepared in dotblot buffer (50 mM Tris, pH 8, 100 mM KCl, 2 mM Mg (OAc)2, 0.5 mM TCEP, 0.0005% NP-40) with a final RNA concentration of 0.1 nM, and Ago2 dilutions from 100 to 0.02 nM. The reactions were incubated at room temperature for 45 min, and then passed through a Protran nitrocellulose and Hybond H+ nylon membrane setup (Cytiva) on a dot blot manifold using vacuum. Blots were used to expose phosphorscreens overnight, followed by quantification using Image Lab (Bio-rad), and plotted with Prism (Graphpad).

## Results

### Role of the miR-106a 3′ end in seedless target recognition

In a previously described miR-CLIP experiment [33] (Fig. 1B), HeLa cells were transfected with sparing concentrations of a pre-miR-106a capture probe, leading to the identification of hundreds of mRNA targets, comprising highly conserved predicted miRNA seed binding sites. We analyzed these targets further and found that they could be subdivided into canonical 8mer, 7mer, and 6mer groups, whereby canonical 8mer targets (*n* = 109) exhibited the strongest repression (with median fold change over targets, fc = −0.42), while more abundant 6mer targets (*n* = 339) were less repressed (fc = −0.11), in accordance with established principles of miRNA targeting (Fig. 1C) [23]. Among the top 1500 most abundant transcripts captured in miR-CLIP, we also identified a “seedless” subset (*n* = 325) that lacked a continuous 6mer seed match between positions A1–C8 of the miR-106a sequence (Fig. 1A), and furthermore, exhibited significant upregulation upon exogenous miR-106a delivery into HeLa cells (fc = +0.11).

To investigate how miR-106a might base-pair with “seedless” targets, we searched the 3′ UTRs of targets for short sequence matches outside the seed region of miR-106a. To validate this approach, we first performed a search for short motifs in canonical miR-106a targets (dark- and medium-blue groups in Fig. 1C). Indeed, as expected, overlapping short motifs of different lengths across the miR-106a seed region (positions A1–U9) were all highly abundant in this target set, which was also significantly repressed (Supplementary Fig. S1). Hence, we performed the same analysis on the 3′UTR sequences of the 325 “seedless” targets using 6mers outside the seed region. We found in 48% of these 3′UTRs 6mers aligning with positions G14–U21 of miR-106a. Three overlapping 6mers (CUGCAC: 77 targets; CCUGCA: 109 targets ACCUGC: 85 targets) appeared most frequently. CCUGCA (U15–G20) was present at least once in 109 of the 325 seedless target mRNAs (Fig. 1D, left panel), which as a group exhibited the highest upregulation (fc = +0.20) after transfection of miR-106a into HeLa cells (Fig. 1D, right panel). These data suggest that several nucleotides in the G14–U21 region of miR-106a contribute to mediating noncanonical interactions with a large proportion of the seedless targets. Note that it should not be interpreted that miR-106a requires exactly this precise 6mer for binding seedless targets, nor that it cannot tolerate bulges or even that it does not require some participation of the seed bases.

To gain further insights into which nucleotides base-pair to the seedless targets, we analyzed predicted base-pairing in sequences surrounding the CCUGCA 6mer in miR-CLIP-captured transcripts (Fig. 1E) and compared it to base-pairing at canonical miR-106a binding sites. Notably, base-pairing predictions were calculated allowing bulges of 1 nt and internal loops of upto 15 nt, but with no other constraints, i.e. no prioritization of seed pairings over other nucleotides, thus representing an unbiased approach for investigating interactions at the nucleotide level that complements the 6mer analysis shown in Fig. 1D.

For sequences surrounding the 7mer seed-match motif in captured canonical targets (*n* = 567) (Fig. 1E, left), we observed the strongest predicted base-pairing across the seed region of miR-106a (positions A2–G8), as expected, with these nucleotides pairing to over 90% of the captured 3′ UTR sequences. The 5′ terminal nucleotide A1 showed minimal complementarity, consistent with its known lack of base-pairing during target recognition [5], whilst U9 contributed to binding in only ∼75% of sequences. Similarly, positions U10–C12, covering part of the central region, exhibited weak base-pairing, while the supplementary (A13–G16) and tail (C17–U21) regions paired with 60%–80% of canonical target sequences. Interestingly, C17–G19 in the tail region displayed notable complementarity, though typically contributing less than canonical interactions. Although these findings align with established miRNA-target pairing rules, they point to strong engagement of the 3′ end of miR-106a during canonical targeting.

In contrast, base-pairing in a 46-nt window surrounding the 6mer CCUGCA (complementary to U15–G20 of miR-106a; *n* = 140) within 3′ UTRs of the seedless targets (Fig. 1E, centre) indicated some weak binding at the junction of the seed and central regions, despite these targets having been selected for having no continuous complementarity to miR-106a seed. Base-pairing with nucleotides G5 to C8 reached 50%–85%; however, it was markedly reduced compared to canonical interactions (>90%). Pairing within the supplementary and tail regions (positions 15–21) was detected in over 90% of seedless sequences, as expected for this set of sequences. It is possible that this extensive 3′ end complementarity can compensate for the absence of a canonical seed match, facilitating stable binding to seedless targets (Fig. 1E, right).

### Seedless target recognition by miR-106a outcompetes other miRNAs

Since the upregulation of seedless targets was observed in HeLa cells transfected with miR-106a probes compared to non-transfected (mock) samples (Fig. 1C), we wondered whether this effect resulted from competitive target site occupancy, where the 3′ end of miR-106a sterically interferes with the binding and repressive action of another miRNA. Indeed, competitive target site interaction between two miRNAs has been previously documented. In one example, the close proximity of the seed-binding sites of miR-184 and miR-205 on the SHIP2 mRNA creates competitive binding on the mRNA target [52]. In another example, closely located seed sites of miR-17 and let-7 on the Bovine Viral Diarrhea Virus RNA might functionally interact to regulate stability of the viral RNA [53]. Whether such competition extends beyond isolated examples remains unexplored.

**Figure 2.**
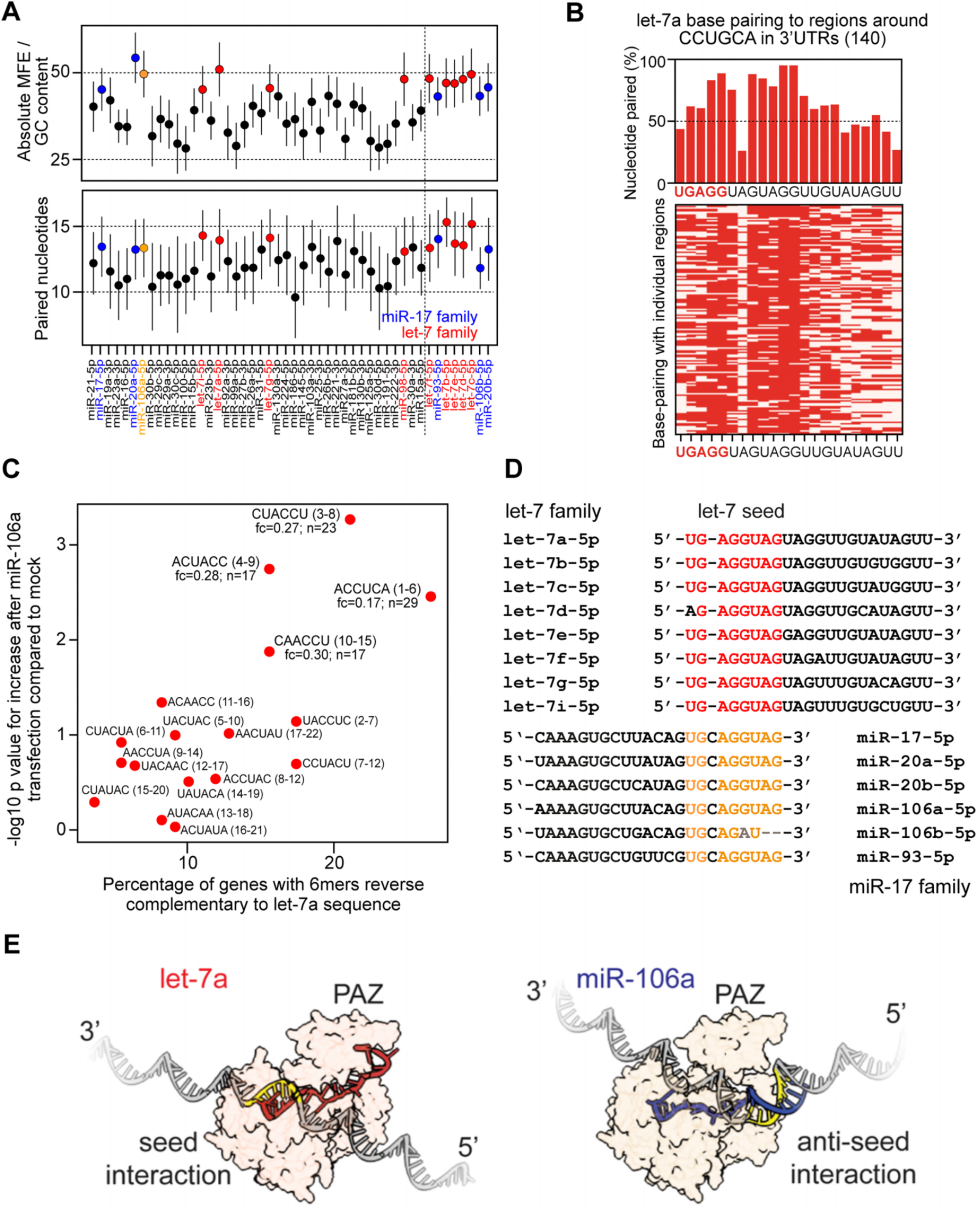
let-7 miRNAs show strong predicted binding to regions around CCUGCA in 3′ UTRs of miR-106a-seedless mRNA targets. (**A**) Absolute MFE divided by GC content of miRNA sequences (top) and number of paired nucleotides predicted by RNAhybrid for the top 40 expressed mature miRNAs in HeLa cells [47], plus additional members of the miR-17 (blue) and let-7 (red) families. Binding was predicted to 140 sequences (46 nt long) surrounding CCUGCA, complementary to the miR-106a 3′ end. Dots represent mean values, with standard deviations shown as vertical lines. (**B**) Predicted base-pairing between let-7a and 140, 46-nt-long sequences surrounding CCUGCA 6mers (as in Fig. 1E). (**C**) Scatter plot showing the percentages of genes with 6mers complementary to let-7a present in the 3′UTRs of 109 CCUGCA-containing genes (see Fig. 1D) plotted against the significance of log_2_ fold change (after miR-106a transfection in HeLa cells, as in Fig. 1C) for groups of genes with let-7a-complementary 6mers in their 3′UTR. The significance was calculated using a two-sided rank-sum test. Notably, for all groups of genes with let-7a-complementary 6mers, the average log_2_ fold change was larger than the one for all genes. Each dot represents a group of genes labeled with the let-7a-complementary 6mer sequence, as well as the corresponding positions of the 6mer in the mature let-7a sequence. For the four most significantly increased groups of genes (*P* < .02; upper right), the average log_2_ fold change and the number of genes are indicated. (**D**) Sequence alignment of miR-17 family members with let-7 family members. (**E**) Cartoon depicting the hypothesized interaction of Ago:let-7a (red guide, 3′ end bound to PAZ pocket) and Ago:miR-106a (blue guide, 3′ end released from PAZ pocket) with an exemplary target based on HMGA2. The cartoon is based on an AlphaFold3 prediction, with the PAZ domain of Ago:miR-106a adjusted for the release effect, based on [54, 55].

To investigate a potential miRNA competition for contested binding sites in the seedless target set, we analyzed the 40 most highly expressed miRNAs in HeLa cells [47] and evaluated their predicted binding strengths. Specifically, we estimated the MFE of binding and the number of paired nucleotides between the complete sequence of each miRNA and its target sequence in a 46-nt window surrounding the most frequent 6mer CCUGCA corresponding to U15–G21 (140 occurences in 109 transcripts; see Fig. 1D). This analysis identified two miRNA families with high binding potential (Fig. 2A).

Members of the miR-17 family, including miR-106a, exhibited high sequence homology, partly due to the tail region, which contains the 6mer complementary to the CCUGCA motif at the center of the 46-nt search window. (Fig. 1E). The MFE analysis predicted strong binding to seedless 3′UTR regions for multiple miR-17 family members: miR-17, miR-20a, miR-106a, miR-93, and miR-20b. The other strongly predicted binder for the seedless sites was the let-7a family, which exhibited a high number of paired nucleotides in the 46-nt search window. We performed the analysis for all let-7 family members, regardless of their expression in HeLa cells, and confirmed that most let-7 miRNA family members, particularly let-7a–e, demonstrate robust binding potential to the seedless target set. We selected let-7a for further analysis due to its high homology with other members of the let-7 family.

### Direct competition between miR-106a and let-7a for let-7 target sites

To identify let-7a nucleotides potentially involved in base-pairing interactions, we analyzed the predicted base-pairing of let-7a to the set of 140 sequences, using the same unconstrained approach as for miR-106a (Fig. 1E). This revealed strong base-pairing for nucleotides G2, A3, G4, G5, U6, and G8 (64%–90% of sequences) of the let-7a seed region, but not for A7 (∼25%) (Fig. 2B). Notably, nucleotides G8–G12 of the central region showed base-pairing (75%–95%) comparable to that of the let-7a seed, suggesting that these residues might also contribute to recognition of the miR-106a seedless targets. The supplementary region (A13–G16) displayed less base-pairing (60%–70%), and most nucleotides in the tail region (A17–G20) exhibited even lower pairing (<50%). Overall, the predicted binding profile of let-7a to miR-106a seedless targets was consistent with both canonical and non-canonical target recognition, while also emphasizing the role of 3′-compensatory interactions, where imperfect seed pairing is reinforced by strong binding contributions from the central and supplementary regions. This aligns with previous findings for let-7 [56], particularly reports that GG dinucleotide (G11, G12) is implicated in enhanced targeting activity [49]. Taken together, analysis of the miR-CLIP-captured seedless target set of miR-106a suggested how the 3′ end of miR-106a could compete with the seed-mediated binding of let-7a.

Next, we determined the abundances of individual let-7a complementary 6mers in the 3′UTRs of the 109 CCUGCA-containing targets from Fig. 1D. We plotted their abundance against their fold change significance after miR-106a transfection in HeLa cells. We found that 6mers representing the let-7a seed region (between positions 1–9) and two other 6mers (starting at positions 7 and 10) were most abundant in these 3′UTRs (>15% each) (Fig. 2C). Furthermore, out of these 6mers, genes with four of them, corresponding to positions 1–6 (ACCUCA), 3–8 (CUACCU), 4–9 (ACUACC), and 10–15 (CAACCU) of let-7a, showed significantly increased expression after miR-106a transfection, with fold changes of +0.17 to +0.30 on average. Together, these four 6mers were found in half of the CCUGCA-containing miR-106a seedless target genes (49.5%; *n* = 54). Overall, this analysis suggested a let-7a target interaction with these genes, compatible with a competitive mechanism where seedless miR-106a binding attenuates canonical let-7 target repression.

Alignment of the let-7 seed region sequence and the 3′ end of miR-106a (Fig. 2D) reveals that both sequences contain a common 6mer AGGUAG, which spans nucleotides A3–G8 of the let-7 family and A18–G23 of the miR-17 family. Furthermore, it is also noticeable that the first two nucleotides of let-7 miRNAs (UG) are identical to nucleotides 15 and 16 of miR-17 family. However, a cytosine at position 17 in the miR-17 family disrupts an extended homology with the let-7 seed region (Fig. 2D). It remains uncertain whether C17 is able to bulge out when binding to target sites of let-7 (*vide infra*), which would be consistent with the flexibility of 3′-end pairing architectures reported previously [49]. For convenience, we refer to the common AGGUAG motif at the 3′ end of miR-106a and the seed region of let-7a as a miR-106a “anti-seed” motif. Of note, this motif is highly similar (one mismatch) to another 6mer, AGGUUG, at position 10–15 of the let-7 sequence, in which the GG dinucleotide is important for let-7 activity [49]. We hypothesize that the two types of interaction, seed or anti-seed, acting at a common exemplary target site, such as HMGA2, lead to two different binding conformations of the Ago:miRNA complex, respectively (Fig. 2E), with potentially different functional outcomes.

### De-repression of let-7a targets

Given the potential for miR-106a anti-seed-mediated de-repression to act broadly on let-7 targets, we conducted further experiments in HEK 293T cells, which have low levels of endogenous miR-106a and are highly transfectable, allowing precise control of miRNA levels with minimal interference from endogenous miRNAs. Furthermore, good-quality data from eCLIP experiments in HEK 293T cells is published [48], revealing the targets of let-7 and miR-17 families captured under native conditions (*vide infra*), and helping to provide independent validation of the aforementioned findings.

**Figure 3.**
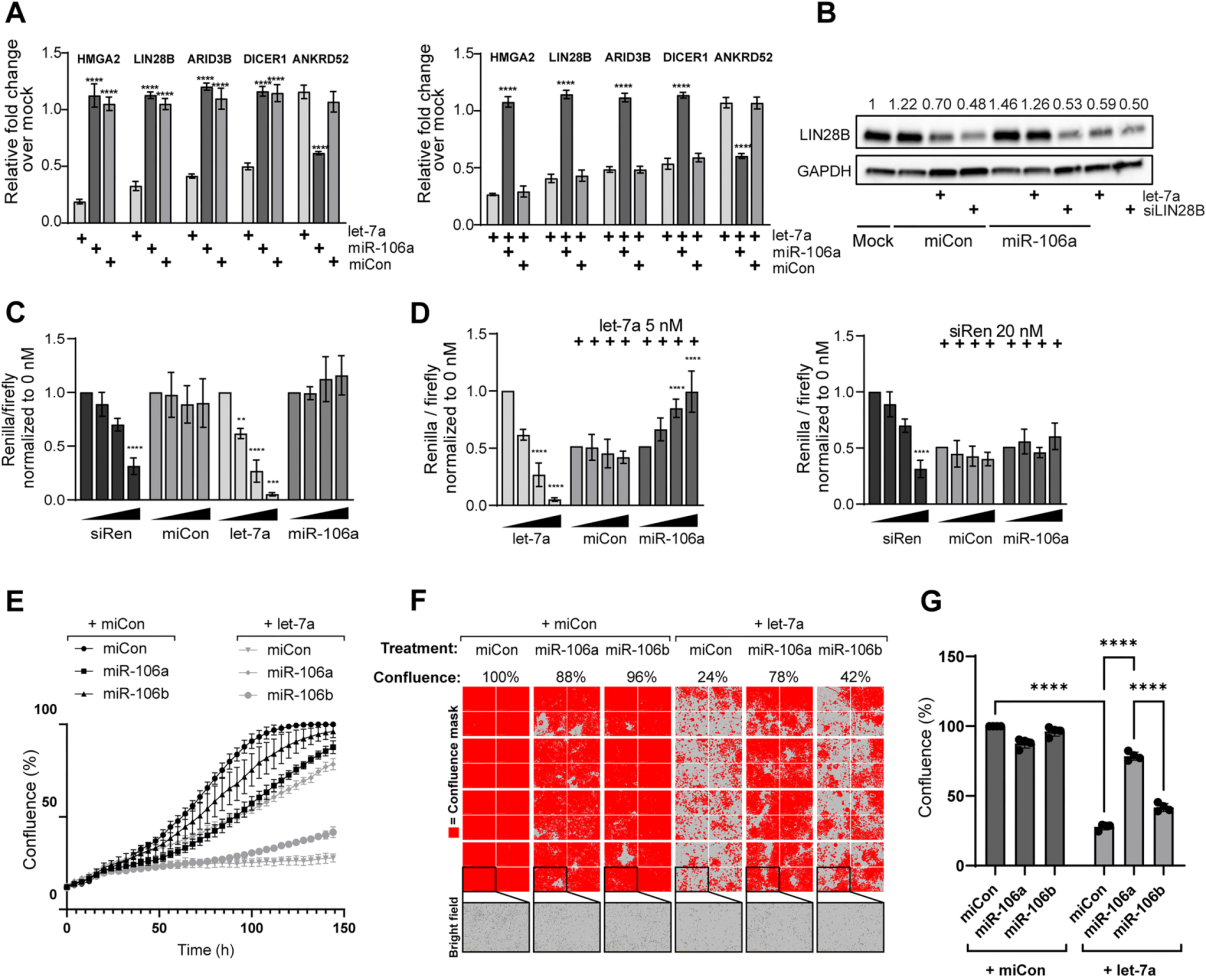
miR-106a competitively counteracts let-7a-mediated repression of validated let-7a targets in cells. (**A**) miR-106a suppresses let-7 repression of endogenous targets. Left panel: Relative fold changes in expression of let-7 targets (HMGA2, LIN28B, ARID3B, and DICER1) and the miR-17 target (ANKRD52) following transfection of HEK 293T cells with 50 nM of let-7a, miR-106a, or negative control miCon. Right panel: Co-transfection of let-7a (1:4 ratio) with either miR-106a or negative control. Fold changes are relative to mock transfections (*n* = 3) and normalized to housekeeping genes ACTB and GAPDH. Statistical significance was determined using two-way ANOVA with Dunnett’s multiple comparisons to gene-specific let-7a single transfections. Error bars indicate SD. ns: not significant; ^****^: *P* ≤ .0001. (**B**) Western blot analysis of the validated let-7 target protein LIN28B and housekeeping control GAPDH in HEK293T cells transfected with 50 nM negative control (miCon) or miR-106a. The let-7a duplex and siLIN28B (positive knockdown control) were transfected at 10 nM either alone or in combination with negative control or miR-106a. Band intensities were normalized to the mock transfection (first lane) and the corresponding GAPDH signal, with normalized values indicated above the blots. (**C, D**) Effect of miR-106a on HMGA2 3′ UTR reporter activity. HEK 293T cells were co-transfected with a reporter plasmid containing the HMGA2 3′ UTR and increasing concentrations (0, 2.5, 10, or 40 nM) of: (C): positive control (siRenilla), negative control (miCon), let-7a, or miR-106a. Significance is shown relative to corresponding concentrations of the negative control. (D) Left panel: let-7a (5 nM) co-transfected with increasing concentrations of either negative control or miR-106a. Right panel: siRenilla (5 nM) co-transfected with increasing concentrations of either negative control or miR-106a. Reporter activity is expressed as *Renilla*/firefly luciferase ratios, normalized to the 0 nM condition. Significance was calculated using a two-way ANOVA with Dunnett’s test. Asterisks denote significance: * (*P* ≤ .05), ** (*P* ≤ .01), *** (*P* ≤ .001), ^****^ (*P* ≤ .0001). *n* = 3. (**E**) Antagonistic effects of miR-106a and let-7a on cancer cell proliferation. Live-cell analysis of DU145 cells transfected 24 h after plating with let-7a, miR-106a, and miR-106b alone or in combination using the IncuCyte® Live-Cell Analysis System. Co-transfection conditions included two miRNAs at 25 nM each for a total of 50 nM. For single-transfection conditions, a negative control miRNA (miR-Con, 25 nM) was co-transfected with the targeted miRNA (25 nM) to maintain the total miRNA concentration at 50 nM constantly. Control samples consisted of only the negative control miRNA (50 nM). Depicted is mean cell confluence as percentage of control-treated cells (miCon) throughout the entire experiment. (**F**) Representative bright-field images and cell confluence areas (red masks) of DU145 cells acquired at 6 days post-transfection with a 10× objective. (**G**) Mean cell confluence as percentage of control-treated cells (miCon) at 6 days post-transfection. Data represent mean ± SD from four technical replicates (*n* = 4). Statistical significance was calculated using two-way ANOVA.

First, we aimed to determine whether the miR-106a-mediated de-repression of let-7 targets observed in HeLa cells could be replicated for a small set of well-characterized let-7 targets upon co-treatment of HEK 293T cells with graded concentrations of miR-106a and let-7a. Therefore, cells were transfected with either a let-7a duplex, miR-106a mimic, a previously described negative control duplex (miCon), or a combination of let-7a and miR-106a. Transcript levels of established let-7a-sensitive genes: *HMGA2, LIN28B, ARID3B*, and *DICER1*, as well as the miR-106a target *ANKRD52*, were quantified via SYBR qPCR. Transfection of let-7a into HEK 293T cells repressed four validated mRNA targets by ~50%–75%, with no effect on *ANKRD52*, as expected (Fig. 3A, left panel). In contrast, miR-106a transfection repressed *ANKRD52* by ~40%, compared to control transfection. Interestingly, this was accompanied by a slight but non-significant increase in let-7a target levels. Importantly, co-transfections of let-7a with miR-106a at a 1:4 ratio led to de-repression of the let-7a targets, resulting in a dramatic upregulation of their expression (Fig. 3A). Control co-transfections with miCon did not affect let-7a-mediated repression, confirming that miR-106a competitively interferes with let-7a function.

To add further support to these findings, we investigated the effects of miR-106a on the regulation of LIN28B protein by let-7a. Single transfections of let-7a duplex or an siRNA targeting LIN28B (siLin28B) markedly reduced LIN28B protein expression, as expected (Fig. 3B and Supplementary Fig. S2). While let-7a exerts its effects by targeting multiple conserved sites in the 3′ UTR, siLin28B acts at a single site in the coding region. Co-transfection of let-7a or siLin28B with miCon did not alter their effects on LIN28B levels. However, co-transfection of miR-106a with let-7a efficiently counteracted let-7a-mediated repression of LIN28B, whereas miR-106a failed to alleviate the inhibitory effect of siLin28B. These results provide strong evidence that miR-106a selectively interferes with let-7a activity on LIN28B but does not affect indiscriminately RNAi-mediated knockdown of the target.

To add further evidence that the effects of miR-106a on let-7 targets resulted from direct physical interactions and not through, e.g. indirect effects on gene expression, we conducted luciferase reporter assays. The full 3′UTR of *HMGA2* was cloned into a dual-luciferase reporter plasmid, and HEK 293T cells were transfected with this construct alongside graded concentrations (0–40 nM) of either miCon, siRen (positive siRNA control duplex, as previously described [57], let-7a mimic, or miR-106a hairpin. Single transfection of the positive control (siRen) produced a dose-dependent suppression of *Renilla* luciferase (Fig. 3C), while the negative miCon was inactive. Also, let-7a suppressed the *HMGA2* reporter, thereby confirming the reporter 3′UTR as a let-7a target. Importantly, miR-106a did not affect the *HMGA2* reporter, strongly suggesting that miR-106a does not independently regulate endogenous *HMGA2 via* its 3′ UTR (*vide infra*).

To assess de-repression of let-7 targets, cells were co-transfected with 5 nM of let-7a duplex and increasing concentrations (0–40 nM) of either miCon or miR-106a mimic (Fig. 3D left panel). In the co-transfection study, let-7a dose-dependently repressed *HMGA2*, and this effect was unaffected by co-transfection with miCon. In contrast, increasing concentrations of miR-106a with let-7a led to dose-dependent de-repression of the *HMGA2* reporter, with full restoration of its expression at 40 nM concentration (Fig. 3D left panel). Importantly, miR-106a did not rescue expression of the siRen-mediated suppression of the same reporter (Fig. 3D right panel), confirming that de-repression is dependent on both let-7a and miR-106a.

Finally, to explore the consequences of anti-seed-mediated antagonism in a functional context, we performed proliferation assays in DU145 prostate cancer cells following transfection with mimics of let-7a, miR-106a, or their combination (Fig. 3E–G). DU145 cells are androgen receptor (AR)-negative, highly aggressive, and treatment-resistant, and they exhibit low endogenous expression of let-7 miRNAs. In cancer cells, LIN28 promotes neoplastic transformation by repressing let-7 miRNAs, which act as suppressors of key oncogenes, including HMGA2 [58]. Consistently, let-7a transfection (25 nM) potently inhibited DU145 proliferation, reducing confluence to 24% compared to miCon treatment (Fig. 3E–G). However, co-transfection of let-7a with miR-106a (1:1 ratio) largely rescued cell proliferation, resulting in 78% cell confluence, comparable to the effect of miR-106a alone (88%) (Fig. 3E–G). This indicates that miR-106a effectively counteracted the antiproliferative effect of let-7a, supporting a functional antagonism through anti-seed-based competition. Consistent with the proposed model of tail seed-based competition, miR-106b—a miR-17 family member carrying a nucleotide change and lacking two terminal nucleotides at its 3′ end (Fig. 2D)—had a much smaller de-repressive effect, yielding only 42% confluence (Fig. 3F).

Taken together, these findings provided additional evidence for a competition-based mechanism between miR-17 family members—specifically miR-106a—and let-7a, where miR-106a effectively prevents let-7a from repressing its targets at both the transcript and protein levels. The reporter assays provided strong support for direct interactions between the miRNAs and their targets, consistent with the miRNA–mRNA cross-linking in the miR-CLIP experiments. Thus, these results suggested that miR-106a might play a broader regulatory role in modulating let-7a activity in cells.

### miR-17 family binds directly to let-7 target sites in mRNA 3′UTRs under native conditions

**Figure 4.**
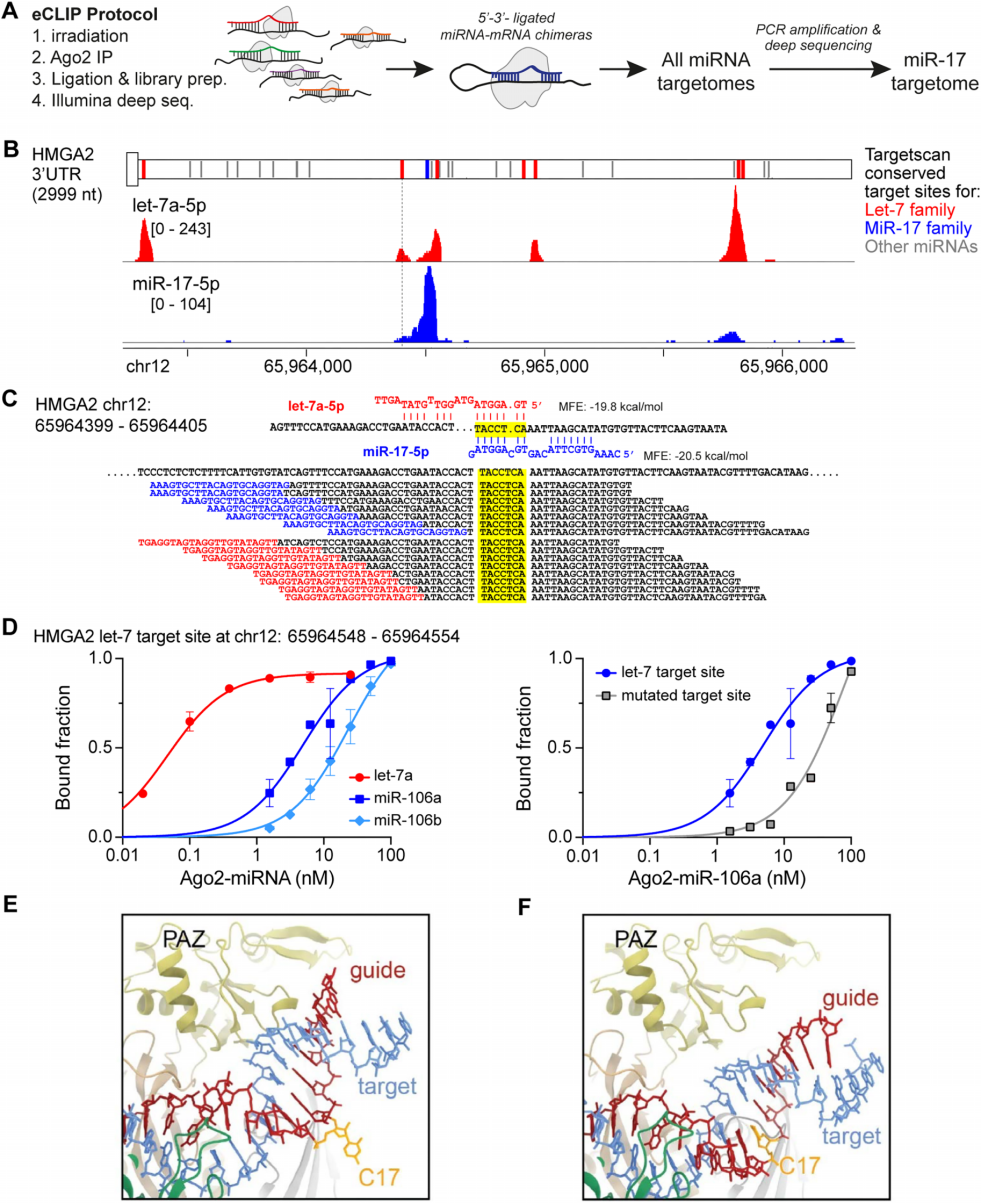
Chimeric eCLIP detects overlapping miR-17 and let-7a reads at predicted let-7a target sites in the HMGA2 3′ UTR. (**A**) Scheme of the chimeric eCLIP method as described by Manakov *et al*. [48]. Ago2–miRNA complexes are cross-linked to target RNA in HEK 293T cells, followed by Ago2 immunoprecipitation and ligation of the miRNA to the target RNA sequence. PCR amplification with miRNA-specific primers enriches for chimeric sequences corresponding to one specific miRNA. (**B**) Genome browser view of eCLIP read densities for let-7a (red) and miR-17 (blue) across the HMGA2 3′ UTR. Maximum read densities are indicated on the left for each miRNA. Predicted miRNA target sites, by TargetScan, [6] are marked as vertical lines in the HMGA2 3′ UTR (top) for the let-7 (red) and miR-17 (blue) families, and for other miRNAs (gray). The regions with predicted let-7 target sites and overlapping eCLIP reads for both let-7a and miR-17 are indicated. (**C**) Pile-up of arbitrarily selected raw eCLIP reads, containing the let-7a (red) and miR-17 (blue) miRNA sequence and their bound target sequences, at the marked region from (**B**). Top: predicted base-pairing using RNAhybrid for let-7a (red) and miR-17 (blue) at each target region. Bottom: Pile-up of 7–8 selected eCLIP reads for miR-17 (upper reads) and let-7a (lower reads). The miRNA sequence part within each chimeric eCLIP read is colored (blue for miR-17 and red for let-7), and the predicted target site sequence within each read is highlighted in yellow. (**D**) Bound fraction as a function of Ago2–miRNA concentration for an HMGA2 target site. Left: Ago2 loaded with let-7a (red), miR-106a (blue), or miR-106b (cyan). Right: Ago2–miR-106a binding to the wild-type target site (blue) and to a mutant (gray) in which nucleotides complementary to the let-7 seed were replaced with adenosines. (**E, F**) AlphaFold 3 predictions of Ago2:miR-106a:anti-seed targets. Two exemplary predictions are shown, highlighting the duplex in the anti-seed region, which stabilizes the release of the miRNA guide from the PAZ pocket (Supplementary Fig. S5).

To demonstrate that miR-17 and let-7 family miRNAs compete for the same binding sites in mRNA 3′UTRs under native conditions, we analyzed publicly available chimeric sequencing data derived from HEK 293T cells using the chimeric eCLIP (enhanced cross-linking and immune-precipitation) method (Fig. 4A). This method captures, miRNA–mRNA chimeras by crosslinking the RISC and subsequently ligating the 3′ end of the miRNA to its target mRNA. Although the ligation step is relatively inefficient and, under some conditions, may be prone to artifacts, it provides transcriptome-wide, state-of-the-art evidence of direct endogenous interactions between a miRNA and its mRNA targets under physiological conditions. eCLIP captures both the miRNA and its mRNA sequence in a single sequencing read, enabling precise mapping of miRNA binding sites while containing information on the identity of the bound miRNA. Recently, Manakov *et al*. reported miRNA–mRNA chimeras for the 94 most abundant miRNAs in HEK 293T cells [48], including miR-17, which differs from miR-106a only at the 5′ terminal nucleotide (Fig. 2D). Since the first nucleotide of a miRNA does not base-pair to the target mRNA and since most chimeric miR-17 eCLIP reads in this published data set curiously lack the first nucleotide, the miR-17 and miR-106a sequences in eCLIP reads are indistinguishable.

We first examined the densities of chimeric eCLIP reads along the 3′ UTR of *HMGA2*, which contains seven conserved let-7 target sites according to prediction by TargetScan (release 8.0) (Fig. 4B). The responsiveness of these sites to let-7 has been previously validated [59], and the aforementioned reporter assay conducted with the complete HMGA2 3′UTR confirmed the strong response to let-7 treatments in HEK 293T cells (Fig. 3). Indeed, high densities of let-7a eCLIP reads were detectable at six of the seven let-7 target sites on the *HMGA2* 3′UTR (Fig. 4B and Supplementary Fig. S3); thus, the predicted (TargetScan) let-7-*HMGA2* interactions are validated by the experimental eCLIP data at these sites. Interestingly, the *HMGA2* 3′ UTR also contains a predicted miR-17 family target site. While increased eCLIP read densities for miR-17 family members were observed at this site, we did not detect repression of *HMGA2* by miR-106a in our reporter assays (Fig. 3C).

Importantly, at four predicted let-7 target sites, we detected chimeric eCLIP reads corresponding to both let-7a and miR-17/miR-106a (Fig. 4B and Supplementary Fig. S3). These data support the presence of competitive binding between let-7a and miR-17 on HMGA2 under native conditions, aligning with the observed de-repression of HMGA2 following miR-106a treatment. Predicted base pairing at these regions consistently indicated canonical seed-mediated binding for let-7a and anti-seed interactions for miR-17 (Fig. 4C and Supplementary Fig. S3).

Notably, at each of the four sites, G2 of let-7a was complementary to a cytidine in the mRNA, completing a canonical seed interaction. This, in turn, positioned a cytidine opposite G16 of miR-17, assuming C17 is unpaired (either stacked in or looped out). Additionally, two of the four sites featured an adenosine opposite U1 of let-7a—a known favorable feature for miRNA–Ago interactions in which the adenosine is bound by Ago protein [5]. This arrangement would produce an A–U base pair at U15 of miR-17, again contingent on C17 being unpaired. Thus, it is unclear whether the interaction with miR-106a results in the adenosine base-pairing to U15 of miR-106a or binding to the Ago pocket.

### *In vitro* binding assays confirm direct engagement of miR-17 family at let-7 target sites

To directly assess whether the observed overlap in eCLIP signal reflects overlapping binding at shared sites, we performed *in vitro* binding assays using recombinant human Ago2 loaded with individual miRNAs and a synthetic RNA fragment corresponding to HMGA2 target site 2 (Fig. 4D, left). All three miRNAs tested, let-7a, miR-106a, and miR-106b, were capable of binding this region, albeit with markedly different affinities. Let-7a exhibited the strongest binding (*K*_D_ = 0.047 nM), consistent with canonical seed-mediated recognition, whereas miR-106a bound with lower affinity (*K*_D_ = 4.9 nM), and miR-106b showed substantially weaker binding (*K*_D_ = 23.3 nM). These data confirm that miR-17 family members can directly engage sequences overlapping validated let-7 target sites, but do so with reduced affinity compared to let-7a, consistent with a binding mode distinct from canonical seed pairing.

To probe the specificity of this interaction, we introduced mutations in the HMGA2 target sequence, replacing the nucleotides complementary to the let-7 seed with adenosines, which simultaneously disrupts the predicted anti-seed pairing for miR-106a (Supplementary Fig. S4). As expected, this mutation strongly impaired let-7a binding (*K*_D_ = 26.5 nM). Similarly, miR-106a binding was also reduced, but the relative reduction in affinity was less pronounced (*K*_D_ = 80.0 nM), suggesting that while the anti-seed region contributes to binding, it is not the sole determinant (Fig. 4D, right). Nevertheless, this reduction further supports a contribution of 3′-end complementarity in the absence of canonical seed. We observed a similar behavior with respect to let-7a, miR-106a, and miR-106b for Dicer target site 2 (Supplementary Fig. S4).

Taken together, these results provide direct biochemical evidence that miR-106a-containing miRISC can bind HMGA2 sequences overlapping let-7 target sites, supporting the model of competitive occupancy. However, the comparatively weaker affinity and incomplete dependence on the predicted anti-seed region suggest that miR-106a binding may involve additional or alternative pairing configurations not fully captured by this minimal *in vitro* system [60]. Moreover, miRNA-target interactions are further shaped by cellular context and Ago regulatory cycles, including phosphorylation-dependent modulation of target binding [61, 62]. Thus, while our *in vitro* assays establish direct binding, they do not fully recapitulate the complexity of miRISC interactions *in vivo*. The precise binding modality of miR-106a at anti-seed sites therefore remains to be resolved. Although co-transfection experiments (Fig. 3) alone do not establish competitive binding, the miRNA-specific derepression observed there, together with eCLIP (Fig. 4B and C) and *in vitro* binding data (Fig. 4D) indicating overlapping target engagement, are consistent with competition between miR-106a and let-7a at shared target sites.

### Structural underpinnings of observed anti-seed-mediated binding

To investigate the structural underpinnings of observed anti-seed-mediated competition, we examined the predicted duplex architecture around C17 at miR-106a binding sites. Structural analyses of RNA duplexes suggest that such bulges can indeed be accommodated without disrupting binding, either by protruding outward (Fig. 4E, similar to PDB ID: 1DQH [63]) or by being integrated within the helix (Fig. 4F, similar to PDB IDL 2JX [64]). To explore the possible impact of such configurations on Ago conformation, we used AlphaFold 3 [50] to model the structure of human Ago2 in complex with miR-106a (Supplementary Fig. S5).

Drawing on these structural predictions, and by analogy with previously characterized target-directed miRNA degradation (TDMD) sites [54, 65], we hypothesize that extensive 3′ pairing at anti-seed sites may induce an open conformation of Ago2. Whether this conformational change impairs downstream silencing—such as GW182 recruitment—remains unclear. Although prior evidence from coding sequence targeting suggests this possibility [21], the Ago2–GW182 interaction involves three binding pockets within the PIWI domain, a region that appears structurally stable across both open and closed Ago2 conformations [5, 65, 66]. These observations raise the possibility that 3′ pairing-induced changes in Ago2 conformation may modulate effector complex formation and contribute to the observed functional antagonism between miR-106a and let-7a. However, while eCLIP analysis identifies binding sites, it does not imply function.

### miR-106a de-represses let-7 targets through a transcriptome-wide anti-seed mechanism

**Figure 5.**
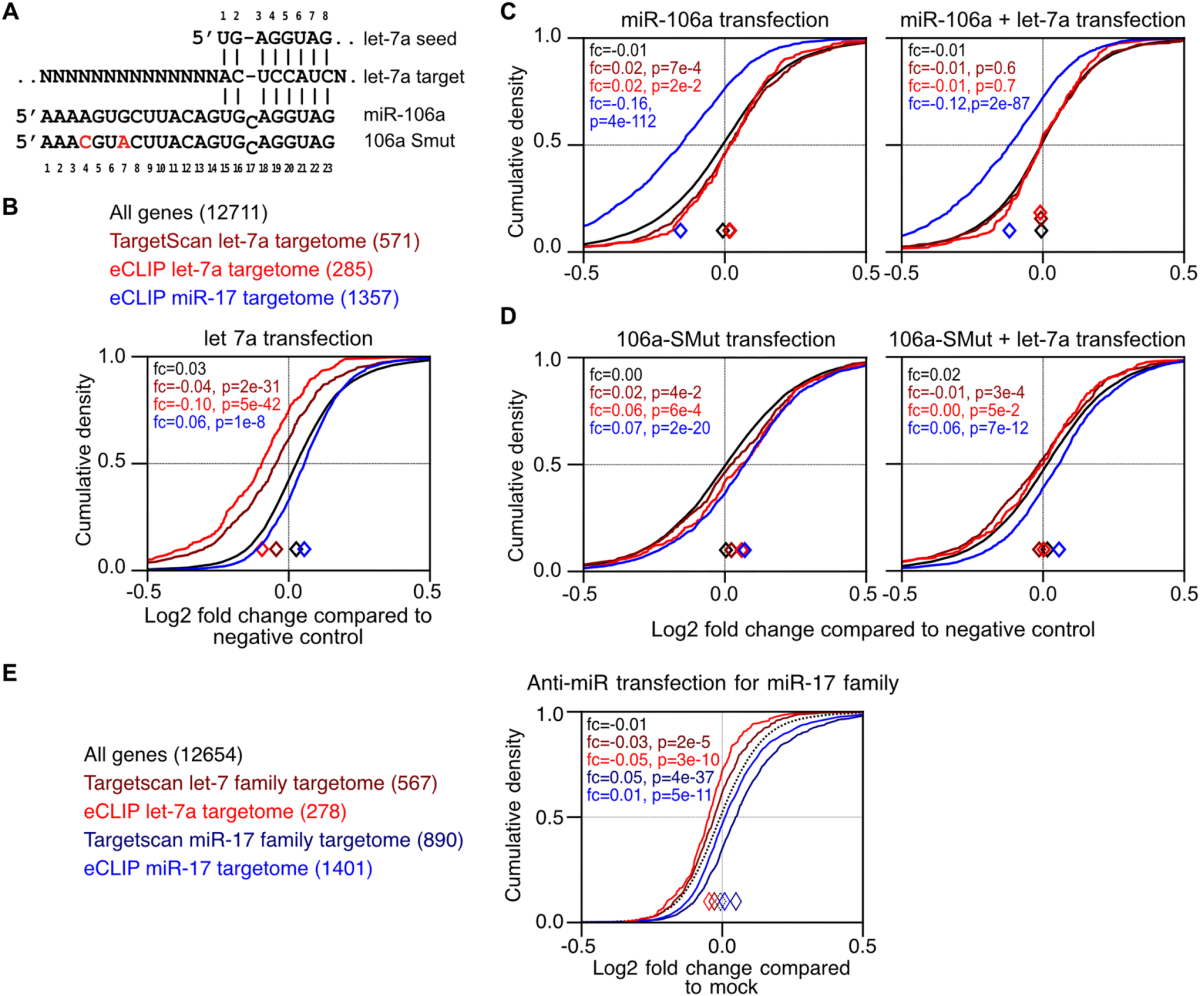
Transcriptome-wide analysis of miR-106a and let-7a interaction in regulating let-7 target gene expression. (**A**) Sequence alignment of the 5p strands of precursor reagents, with mismatches relative to miR-106a highlighted in red. (**B**) Cumulative density function (CDF) plot of log_2_ fold changes in gene expression in HEK 293T cells following let-7a transfection compared to a negative control transfection. Each CDF curve represents one of four gene groups: all genes (black), genes with TargetScan-predicted sites for the let-7 family but not the miR-17 family (dark red), genes with eCLIP peaks for let-7a in the 3′ UTR but not for miR-17 (red), and genes with eCLIP peaks for miR-17 in the 3′ UTR but not for let-7a (blue). Median fold changes for each gene group are marked by diamonds. *P*-values indicate statistical significance for comparisons with the distribution of all genes, calculated using a Wilcoxon rank-sum test. Leftward shifts reflect stronger target downregulation (C–D) CDF plots as in panel (B), but following transfection with: (**C**) miR-106a alone (left), or co-transfection of miR-106a with let-7a (right), (**D**) a miR-106a seed mutant with two nucleotide changes at positions 4 and 7 (left), or the same mutant co-transfected with let-7a (right). (**E**) CDF plots as in (Fig. 5B), but for log_2_ fold changes after transfection with anti-miRs targeting four miR-17 family members: miR-17, miR-20a, miR-93, and miR-106a. In addition to the four groups of genes shown in panel (B), predicted target genes of the miR-17 but not the let-7 family are shown (dark blue). All genes are shown as a dashed line.

To further explore the anti-seed activity and its implications for the let-7a targetome, we performed a transcriptome-wide analysis of gene expression changes in HEK 293T cells upon single transfections of pre-miRNA hairpins and co-transfections of miRNAs hairpins together with let-7a duplex. First, HEK 293T cells were independently transfected with let-7a duplex, negative control miCon, miR-106a, or miR-106 seed mutant (106a-Smut) in which two positions of the seed were mutated (Fig. 5A). The 106a-Smut was designed to assess the overall influence of the seed region on de-repression of let-7 targets, and also, to evaluate de-repression independently of canonical miR-106a activity. Expression analysis was performed on: i) genes comprising chimeras of let-7 and miR-17 miRNAs in eCLIP data, and ii) predicted targets of miR-17, let-7 and miR-16 (as a control) based on TargetScan.

Transfection of let-7a into HEK cells resulted in significant downregulation of its targets from both chimeric eCLIP clusters (fc = −0.1) and TargetScan-predicted sites (fc = −0.04), compared to negative control transfection (miCon) (Fig. 5B and Supplementary Fig. S6). Likewise, miR-106a transfection suppressed both chimeric miR-17 eCLIP targets (fc = −0.16) and those predicted by TargetScan (fc = −0.22). Furthermore, it produced a modest but significant upregulation of eCLIP (fc = +0.02) and TargetScan (fc = +0.02) let-7a targets, consistent with attenuation of baseline activity of endogenous let-7a (Fig. 5C).

Given that the single transfection data aligned with our main hypothesis of an anti-seed interaction, we anticipated that co-transfection would amplify the observed effects. Indeed, co-transfection of miR-106a (50 nM) with let-7a (10 nM) (Fig. 5C) resulted in robust de-repression of both predicted and chimeric let-7 eCLIP targets. These findings confirmed the impact of miR-106a on let-7a function. Notably, co-transfection of 106a-Smut (50 nM) with let-7a (10 nM) (Fig. 5D) resulted in de-repression of both eCLIP (fc = 0.00) and TargetScan (fc = −0.01) let-7a targets. Overall, these findings confirmed that miR-106a-induced de-repression of let-7 activity occurs across a wide range of predicted and experimentally validated let-7 targets. Furthermore, the miR-106a seed appeared unnecessary for this activity, since the seed mutations did not attenuate the effect.

Notably, miR-20a, miR-93, and miR-17, which are reportedly expressed at higher concentrations than miR-106a in HEK 293T cells [48], share identical anti-seed sequences with miR-106a (Fig. 2D), and thus would be expected to contribute to a miR-17 family anti-seed activity. Thus, we tested if blocking all endogenous anti-seed-containing miR-17 family members by transfecting cells with commercially available synthetic anti-miR reagents would affect let-7 target genes. An equimolar combination of anti-miRs directed to miR-17, miR-106a, miR-93, and miR-20a was produced and characterized in HEK293T cells by co-transfection with luciferase reporter constructs comprising either a miR-106a sensor (fully complementary to miR-106a) or a HMGA2 target site T6. (Supplementary Fig. S7). The antimiR-17 family produced a robust de-repression of luciferase when transfected with the miR-106a sensor, presumably through inhibition of endogenous miR-17 family. In contrast, when co-transfected into cells with the HMGA2 T6 reporter, a statistically significant inhibition of luciferase was observed, presumably due to release of endogenous let-7 activity (Supplementary Fig. S7). We treated HEK293T cells with the antimiR-17 family and conducted a transcriptome-wide analysis of gene expression changes. We observed a significant repression of predicted (TargetScan) and experimental (eCLIP) let-7 target genes (Fig. 5E), consistent with an increased let-7 targeting due to the absence of miR-17 anti-seed binding competition. In contrast and as expected, predicted and experimental miR-17 target genes significantly increased upon transfection with miR-17 family anti-miRs. These results strongly support a competitive binding hypothesis between let-7a and miR-17 family, including miR-106a.

### Mapping the competitive interactome between miR-106a and let-7a through shared, regulated binding sites

**Figure 6.**
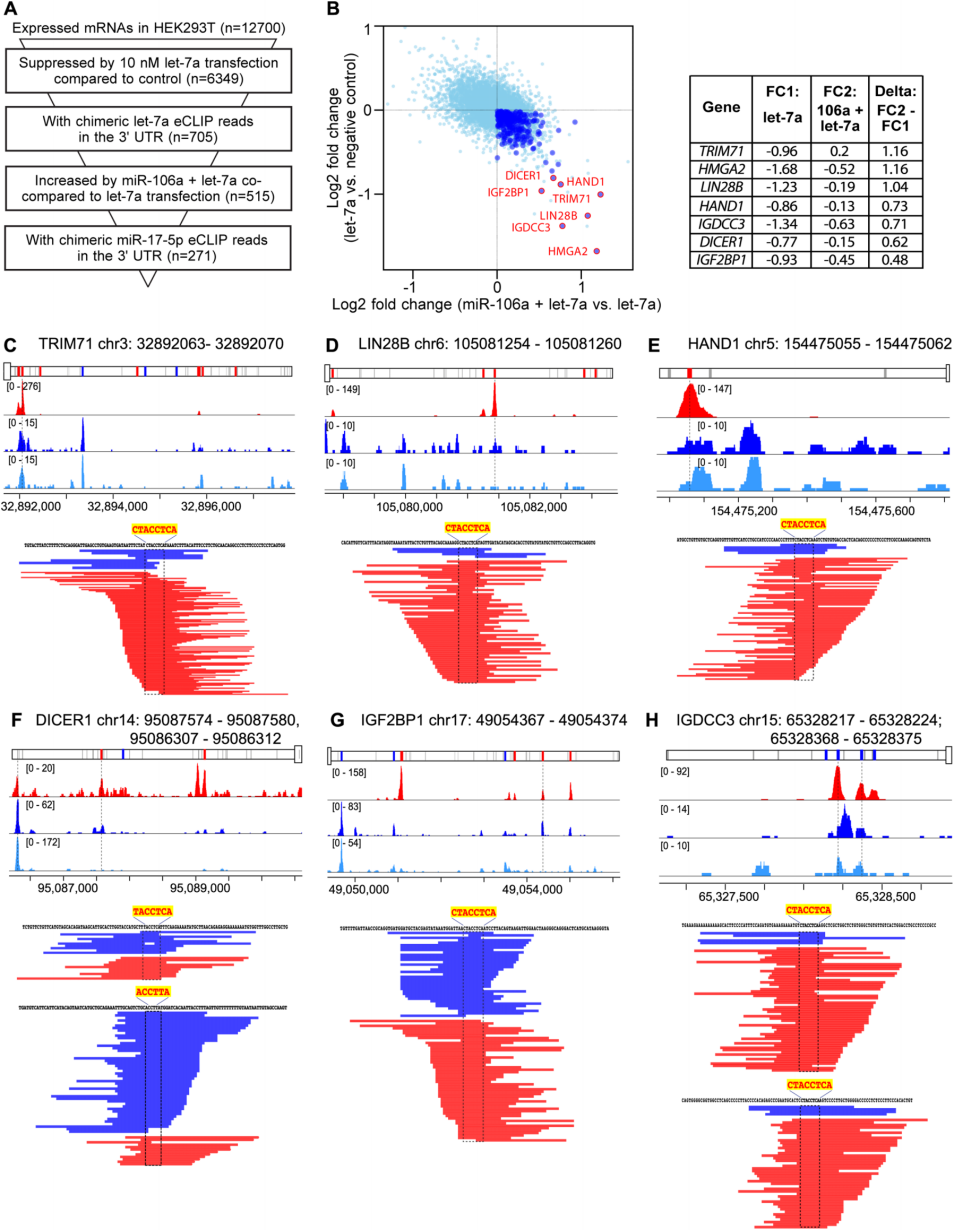
Chimeric eCLIP reads for let-7a and miR-17 overlap in the 3′ UTRs of genes in HEK 293T cells. (**A**) Selection of candidate genes with eCLIP peaks for both let-7a and miR-17 in their 3′ UTRs, and with fold changes that are lower after let-7a transfection and higher after miR-106a/let-7a co-transfection in comparison to the median fold change for all genes. (**B**) Left panel: scatter plot of log_2_ fold changes after let-7a transfection (*y*-axis) compared to let-7a + miR-106a co-transfection compared to let-7a single transfection (*x*-axis). Dark blue dots represent filtered genes with: (i) log_2_ fold changes lower than the median after let-7a single transfection compared to the control, (ii) log_2_ fold changes higher than the median after let-7a + miR-106a double transfection compared to let-7a single transfection, (iii) a let-7a eCLIP peak in the 3′ UTR, and (iv) a miR-106a eCLIP peak in the 3′ UTR. Other genes are shown in light blue. Seven strongly responding genes shown in Figs 4 and 6C–H are labeled in red. Right panel: Table with the following log_2_ fold changes for the seven labeled genes: let-7a single transfection compared to control (FC1), let-7a + miR-106a double transfection compared to control (FC2), and let-7a + miR-106a double transfection compared let-7a single transfection (FC2-FC1, “delta”). (**C**–**H**) Top panels: Genome browser views (similar to Fig. 4B), showing densities of chimeric eCLIP reads for let-7a (red) and miR-17 (blue). The maximum eCLIP read densities are indicated at the left for each miRNA. Bottom panels: Pile-ups of arbitrarily selected eCLIP reads for miR-17 (blue) and let-7a (red) at predicted (by TargetScan) and a non-predicted [lower site in panel (F)] let-7 target sites in the 3′ UTRs of six selected genes. The number of reads shown was adjusted to fit in the figure borders, by randomly removing reads.

To define the effective competition interactome of miR-106a and let-7 in HEK 293T cells, we filtered the genes based on eCLIP binding data and their behavior in miRNA transfection experiments (Fig. 6A). We first identified genes that decreased more than the median across all genes after let-7a transfection. From this group, we extracted 705 genes that comprised let-7a eCLIP reads in their 3′UTRs, 515 members of which were increased by co-transfection of miR-106a and let-7a. Greater than 50% of the genes in this subset (271) had chimeric eCLIP peaks in their 3′ UTR for both let-7a and miR-17 (Fig. 6A), including 31% with overlapping eCLIP peaks (Supplementary Table S5). Ranking these genes by their de-repression fold change (difference between miR-106a and let-7a co-transfection and single let-7a transfection), revealed *HMGA2, LIN28B*, and *DICER1* among the top responders (Fig. 6B), aligning with the data from the cellular assays (Fig. 3).

We performed a similar analysis as for *HMGA2* (Fig. 4B and C), with six additional top-ranked de-repressed genes from the interactome (Fig. 6B), analyzing reads at eight arbitrarily chosen miR-17/let-7a contested sites. All these sites overlapped with TargetScan-predicted target sites for let-7a. The analysis confirmed that chimeric eCLIP read densities for both let-7a and miR-17 were concentrated around a common 8mer, CTACCTCA, which is complementary to nucleotides 1–8 of the let-7a seed. This motif is also complementary to the six nucleotides 18–23 (CTACCT) of the anti-seed region of miR-17 or, in 7/8 of the selected cases, complementary to eight 3′ nucleotides of miR-17 where C17 is not base paired (Fig. 6C–H).

The genome browser representations in Fig. 6C–H show that overall, let-7a-associated eCLIP reads are more abundant than those of miR-17 at most sites; some targets, such as *IGF2BP1*, exhibit more balanced read densities. A similar pattern was observed for *DICER1* (Fig. 6F), where we analyzed two sites in the 3′ UTR. Notably the site closest to the start of the 3′ UTR, which is not a predicted TargetScan let-7 site, attracted more miR-17 than let-7a reads. This may be due to a shorter, 6-mer let-7 seed match that includes a G-U wobble base pair, possibly resulting in weaker let-7a binding compared to the other example genes.

Overall, this analysis provided further strong evidence for a competitive interactome between miR-106a and let-7a in HEK 293T cells, in which miR-106a mitigates the repression exerted by let-7a on its mRNA targets. The identification of common eCLIP binding sites in 3′UTR regions with complementarity to the let-7a seed and miR-17 anti-seed in native HEK 293 cells further supports the hypothesis of an anti-seed mediated de-repression and extends it to a broad range of targets.

### Exploring the architecture of 3′ end sequence for miR-106a anti-seed function

**Figure 7.**
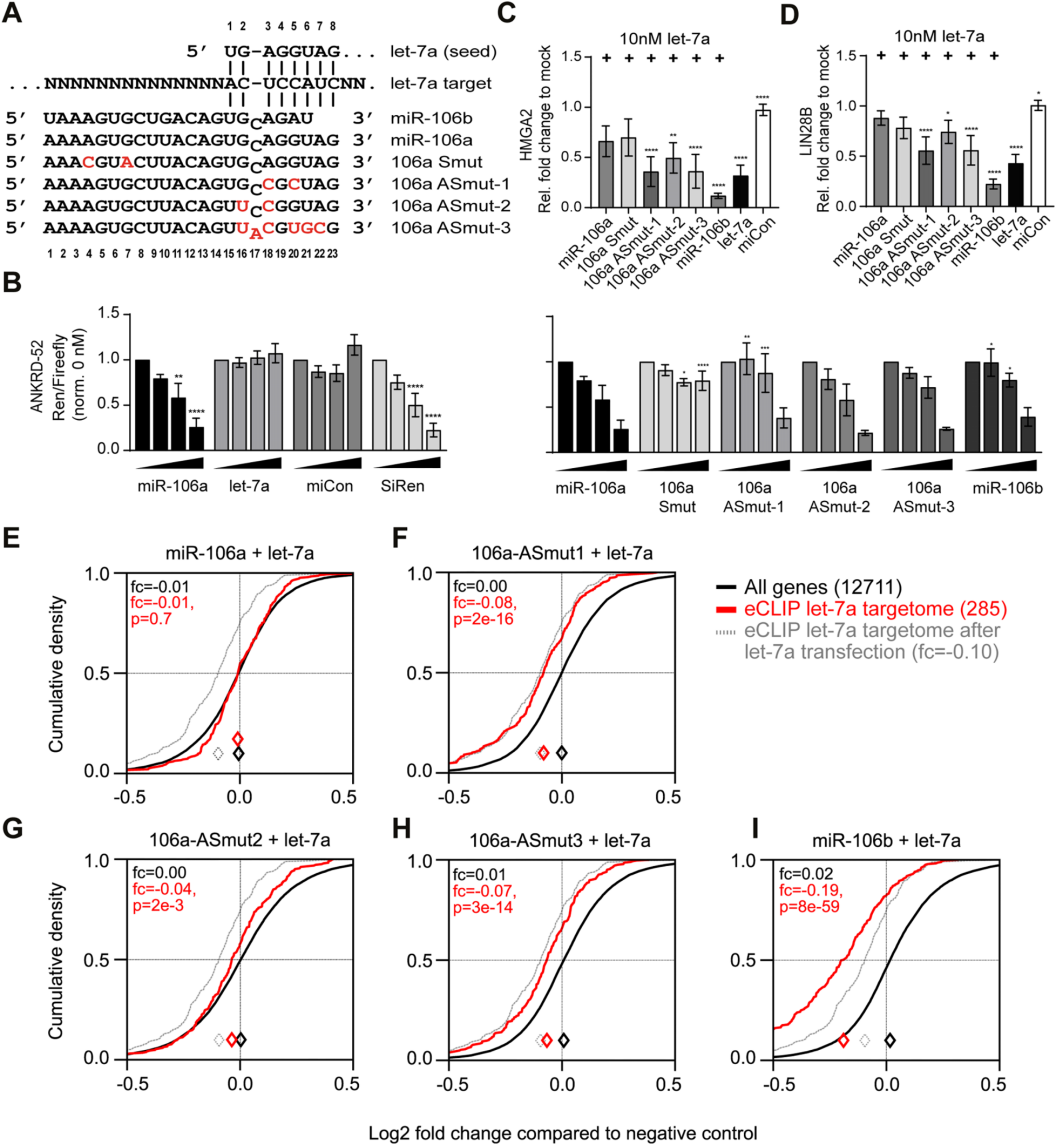
Validation of mutant miRNAs and transcriptome-wide investigation of the function of the miR106a antiseed sequence in HEK 293T cells. (**A**) Sequence alignment of the 5p strands of precursor reagents, with mismatches relative to miR-106a highlighted in red. (**B**) Relative luminescence of the ANKRD52 3′ UTR luciferase reporters in HEK 293T cells. Transfections were performed with increasing concentrations (0, 2.5, 10, or 40 nM) of siRenilla (positive control), let-7a duplex, randomized negative control duplex (miCon), or miR-106a, miR-106b, seed-mutated miR-106a, and 3′ end-mutated miR-106a mutants #1–3. *Renilla* luminescence was normalized to Firefly luminescence and expressed relative to mock transfections (*n* = 3, set to 100%). Statistical significances from a two-way ANOVA with Dunnett’s multiple comparisons to respective 0 nM treatment (left) or miR-106a (right) are shown on the graph. Error bars represent SD. ns, not significant (omitted). *: *P* ≤ .05; **: *P* ≤ .01; ***: *P* ≤ .001; ^****^: *P* ≤ .0001. (**C, D**) Relative fold changes of endogenous let-7 targets, HMGA2 (left) and LIN28B (right) following transfection of HEK 293T cells with 50 nM randomized negative control duplex (miCon), 10 nM let-7a duplex alone, or co-transfected with 50 nM of one of the following: miR-106a, miR-106b, seed-mutated miR-106a (Smut), or three miR-106a 3′ end mutants (ASmut-1, -2, -3). Fold changes are normalized to mock transfections (*n* = 4) and further standardized using housekeeping genes ACTB and GAPDH. Statistical significances from a two-way ANOVA with Dunnett’s multiple comparisons to miR-106a are shown on each graph. Error bars indicate SD. ns, not significant; *: *P* ≤ .05; **: *P* ≤ .01; ***: *P* ≤ .001; ^****^: *P* ≤ .0001. (**E**-**I**) CDF plots of log_2_ fold changes in gene expression following miRNA transfections in HEK 293T cells compared to negative control transfections, for miRNAs with specific nucleotide mismatches at the miR-106a 3′ end. Each panel represents a co-transfection of let-7a duplex with miR-106a (E), ASmut-1 (F), ASmut-2 (G), ASmut-3 (H), and miR-106b (I). Cumulative densities are shown for two groups of genes: all genes (black), and genes with eCLIP peaks for let-7a in the 3′ UTR and not for miR-17 (red). The dotted gray line shows as a reference, for the second (red) group of genes, the distribution of fold changes after single let-7a transfection compared to negative control transfection. Median fold changes are marked by diamonds and indicated inside each panel together with the *P*-value for comparisons of fold changes with those of all genes from a Wilcoxon ranksum test. Leftward shifts reflect stronger target downregulation.

To gain further insight into the role of the 3′ end of miR-17 family members in anti-seed activity, we turned to miR-106b. According to miRBase (www.miRBase.org), miR-106b has a tail region that is two nucleotides shorter than that of other miR-17 family members and carries a G→A substitution at position 20 (Fig. 2D). While this truncation is unlikely to affect canonical seed interactions, it would be expected to reduce coverage of the let-7 seed region in let-7-targeted mRNAs. To probe this further, we generated three synthetic variants of miR-106a bearing specific alterations to its 3′ end (Fig. 7A) and examined their targeting properties in comparison to miR-106a.

The anti-seed mutant 1 (ASmut-1) contained A18→C18 and G20→C20 substitutions, extending the putative C17 bulge toward the 3′ end and disrupting the G19–G20 motif implicated in stabilizing supplementary base pairing [49]. ASmut-2 carried G16→U16 and A18→C18 substitutions, further compromising complementarity around C17. ASmut-3 introduced six mutations that effectively eliminated the original 3′ context of the miRNA. We transfected each variant in its precursor hairpin form, along with wild-type miR-106a and miR-106b hairpins, into HEK 293T cells and performed small RNA sequencing. All variants were processed into the expected mature miRNAs with high fidelity, with only small numbers of *iso*-miRs. Notably, a large number of reads corresponding to a 1-nt longer *iso*-miR of miR-106b were also detected (Supplementary Fig. S8).

Next, we confirmed the functionality of the miR-106a variants using a luciferase reporter comprising the *ANKRD52* 3′UTR (Fig. 7B). In single transfections, miR-106a, miR-106b, and siRen showed strong, concentration-dependent inhibition of luciferase, whereas miCon, let-7a, and 106a-Smut were inactive. Both miR-106b and the anti-seed mutants showed similar levels of reporter suppression, albeit slightly less than that of miR-106a. The data confirmed that in addition to being properly processed, the miRNAs are all able to suppress a canonical miR-17 target.

We then assessed the performance of the reagents using qRT-PCR on let-7 mRNA targets *HMGA2* and *LIN28B*. Consistent with earlier observations on the let-7 transcriptome (Fig. 4), co-transfection of miR-106a with let-7a significantly reduced the let-7a-repression of *HMGA2* (Fig. 7C) and *LIN28B* (Fig. 7D) compared to let-7a single transfections. As expected, the seed-mutated miR-106a also attenuated let-7a-mediated repression of *HMGA2* and *LIN28B*. However, mutations at the 3′ end of miR-106a impaired its de-repressive activity, with ASmut-1 and ASmut-3 showing comparable hindrance, while ASmut-2 was less effective at reducing the anti-seed effect of miR-106a. Unexpectedly, co-transfection of let-7a with miR-106b enhanced the repression of let-7a on both targets, indicating a potential synergistic effect between the two miRNAs, which we cannot yet explain.

Finally, as performed for miR-106a (Fig. 5), we conducted single- and double-transfections of the miR-106a variants with let-7 and measured the effects on the 285 targets of the let-7a eCLIP targetome. In co-transfections with let-7a, ASmut-1 and ASmut-3 strongly impaired the anti-seed activity (fc = −0.08 and fc = −0.07, respectively) of miR-106a (fc = −0.01) to a similar degree (Fig. 7E–H), despite the three additional mutations in ASmut-3. On the other hand, compared to miR-106a, ASmut-2 exerted only a mild effect on let-7a activity (fc = −0.04), despite having the same number of point mutations as ASmut-1, albeit shifted toward the miRNA supplementary region. Together, these observations suggested that for anti-seed activity, the placement of mismatches is more important than their number.

Surprisingly, co-transfections of miR-106b with let-7a enhanced the downregulation (fc = −0.19) of let-7a targets (Fig. 7I), surpassing the repression observed with let-7a transfection alone (fc = −0.1). This result was consistent with the aforementioned effects on *HMGA2* and *LIN28B* (Fig. 7C and D, respectively). We surmise that the enhanced repression involves factors beyond anti-seed interactions.

A complementary way to assess the functional relevance of the miRNA competition, is to examine the evolutionary sequence conservation at competition sites. Indeed, predicted let-7 target sites in de-repressed genes exhibited significantly higher conservation scores downstream, the region where supplementary or partial seed interactions with miR-17 may occur, than other, not derepressed let-7 target sites (Supplementary Fig. S9). Notably, there was no difference in conservation scores upstream of let-7 target sites or within these sites, where conservation scores are highest.

## Conclusions

The discovery of miRNAs heralded intense efforts to define the complete miRNA repertoire and their targetomes across species [1]. According to miRBase v22 [39], over 2600 distinct human miRNAs have been annotated, with many grouped into conserved seed families [23]. Canonically, miRNAs function as integral units within Ago protein complexes [11], where the seed region (typically nucleotides 2–8) serves as the primary determinant for target binding [2, 67]. Structural studies have shown that the 5′ half of the seed is solvent-exposed within Ago:miRNA complexes [11], enabling a conformational change that extends pairing across the full seed and into the supplementary region [11, 20].

Parallel targeting by multiple miRNAs at distinct seed sites within a 3′ UTR is a founding concept in miRNA biology [68]. Cooperative effects can arise when miRNA sites are appropriately spaced [7, 69]. In contrast, when target sites overlap or are too closely located, miRNAs may interfere with each other’s binding, as exemplified by miR-184 blocking miR-205-mediated suppression of SHIP2 mRNA through overlapping seed-binding regions without itself repressing the target [52].

Our objective in this study was to clarify how a subset of mRNAs was covalently captured by miR-106a using a novel miR-CLIP method [33]. The experiments revealed a distinct form of competition between two prominent miRNA families; miR-17 and let-7, at shared binding sites. The data reveal that miR-106a, a member of the miR-17 family, antagonizes let-7a function via an unusual “anti-seed” interaction, wherein nucleotides of the 3′ tail engage the target independently of canonical seed pairing. This tail sequence is highly conserved among miR-17 family members, with the notable exception of miR-106b. *In vitro* binding assays provided evidence for overlapping targeting of shared sites between miR-106a and let-7a, further confirmed at endogenous levels by the analysis of chimeric eCLIP datasets [48], which revealed a multitude of chimeric reads ligated to both miR-17 and let-7 family members at common regions.

Functional experiments comparing miR-106a, miR-106b, and synthetic anti-seed mutants further established that only miR-106a could antagonize let-7-mediated repression at selected 3′ UTR sites. In contrast, miR-106b, differing in both tail sequence and length, appeared to enhance let-7 repression, possibly through cooperative binding at adjacent sites stabilized by TNRC6 interactions [7, 69]. Although speculative, this suggests that subtle differences at the 3′ end of miRNAs can shift the outcome between competition and cooperativity depending on context. Specifically, we suspect that miR-106b does not lead to an extended anti-seed duplex, fails to prevent let-7 binding, and is thus similar to previously reported seedless sites which require cooperativity [70].

Importantly, the observed antagonism was not merely biochemical but translated into functional consequences. De-repression of key oncogenic let-7 targets, such as HMGA2 and LIN28B, and rescue of proliferation in DU145 prostate cancer cells by miR-106a, highlight the potential physiological relevance of anti-seed-mediated competition. Such effects suggest that miRNA:miRNA antagonism may constitute an additional regulatory layer in cancer biology.

Beyond canonical interactions, emerging evidence highlights the relevance of seedless target recognition in miRNA function. Several studies have characterized seedless binding sites that either require cooperative contexts [70] or operate through alternative mechanisms such as steric hindrance of ribosomal translation [21]. While these seedless sites typically fail to repress efficiently on their own, our data show that extensive 3′ end pairing, combined with partial or no seed involvement, can nonetheless exert functional effects via competitive binding, rather than direct repression.

Recent studies investigating 3′-only sites for miR-155 and miR-148 [71] further support that extended 3′ pairing can mediate strong Ago binding, though efficient repression generally requires additional seed pairing. Our anti-seed targets resemble these sites, combining strong 3′ interactions with minimal seed engagement, and may represent a special class of functional, slow-dissociating complexes that modulate miRNA competition.

Structural considerations provide further insights. Predictions based on AlphaFold3 modeling of the Ago2:miR-106a complex, together with analogies to structures of TDMD-inducing sites [54, 65], and especially recent fully complementary targets, [54, 55], suggest that extensive 3′ pairing may favor an open Ago2 conformation. Although GW182 binding sites within the PIWI domain are structurally preserved between conformations [11, 66], spatial reorganization induced by extended 3′ complementarity might nonetheless hinder GW182 recruitment or its functional coupling, thus favoring de-repression without full RISC activation. Indeed, the previous study of steric hindrance of ribosomes by seedless sites reported that while such sites were able to recruit Ago, they did not recruit GW182, which required seed binding [21].

Notably, the miR-17 anti-seed region harbors a cytidine at position 17 that interrupts an extended match (positions 15–23) with the let-7 seed region (nucleotides 1–8). While let-7 offset-6mer sites (positions 3–8) enable gap-free targeting by the miR-17 anti-seed, sites extending to positions 1 or 2 require a C-bulge in the miR-17 sequence, whereas perfectly complementary anti-seed sites would instead require a bulge in the target. Analysis of eCLIP reads from seven strongly de-repressed target mRNAs (Figs 4 and 6) showed that 10 of 12 target sites were complementary to U15-G16 of miR-17, consistent with the presence of a C-bulge. Structural studies indicate that such bulges can be accommodated without major loss of duplex stability, permitting extended pairing beyond isolated mismatches [63, 64]. At present, it remains unclear whether bulge formation is important for derepression or whether pairing of miR-106a terminates prior to this position. One possibility is that a bulged nucleotide in either miRNA or target sequence affects how the two miRNAs engage shared targets, thereby contributing to the observed competition outcome (let-7 seed binding and target repression versus miR-17 anti-seed binding and no target repression).

It is tempting to speculate that tail–seed overlaps may represent a widespread feature embedded in miRNA evolution. To assess this possibility, we analyzed ∼1500 unique miRNA 6mer seed sequences from miRBase, alongside their reverse and shuffled seed controls. Over 95% of real seeds had a match to a 3′ end of another miRNA (starting from nucleotide 10; Supplementary Table S6), compared to ∼91% of reversed seeds (*P* < 1e-3) and ∼90% of shuffled sequences (*P* < 1e-8) (Supplementary Fig. S10). Of these, real seeds had significantly more 3′end matches (with ∼6.8 unique 3′end sequences, on average) than reverse (∼5.3) or shuffled (4.8) controls (*P* < 1e-21). These findings indicate that short overlaps between miRNA 3′ ends and seed motifs are more common than expected by chance, raising the possibility that additional miRNA families may participate in similar competitive interactions via noncanonical 3′ pairing. While such overlaps occur at the sequence level and are enriched over random expectation, they may provide a basis for functionally relevant interactions in specific contexts. These configurations may be particularly important where miRNAs co-target shared transcripts and act antagonistically, allowing small imbalances in activity to be amplified through competition and leading to threshold-like shifts in regulatory output and competing regulatory states.

The miRNA competition described here represents an unusual mechanism by which mRNAs are protected from let-7 mediated repression and thereby stabilized. We cannot exclude the possibility that other factors such as RBPs may also play a role. Several RBPs are known to bind and stabilize mRNAs [72, 73], and members of the IGF2BP family in particular have been reported to bind near structured regions surrounding let-7 mRNA target sites and inhibit let-7 activity [74]. Further experimental work will be required to disentangle the potential contributions from other factors.

Our findings also carry implications for therapeutic strategies targeting miRNAs. While miRNAs represent an under-developed, versatile, and attractive new class of oligonucleotide drug targets, full success in clinical trials has not yet been realized [22]. Farabursen is a chemically modified oligonucleotide targeting miR-17 that is efficacious in models of autosomal dominant polycystic kidney disease [75, 76], and is currently in clinical testing (NCT05521191). Since the miR-17 family comprises six members from three genomic clusters, full complementarity to all family members is unfeasible. Farabursen therefore targets only 9 nt at the 5′ end of miR-17, including the seed, with the expectation that shared seed sequences will ensure functional co-repression of the entire miR-17 family. However, our results, demonstrating distinct and even opposing functions of miR-106a and miR-106b driven by their divergent 3′ ends, suggest that therapeutic targeting based solely on seed identity may overlook critical noncanonical contributions and oversimplify functional redundancy within miRNA families.

In summary, our findings uncover an unexpected mode of competition between miR-17 family members and let-7, mediated by extensive 3′ end interactions. They highlight the regulatory importance of noncanonical 3′ pairing, demonstrating that subtle sequence differences at the miRNA 3′ end can fundamentally alter competitive outcomes among miRNA family members. More broadly, they reveal an additional layer of complexity in miRNA-mediated regulation, where interactions beyond the seed region not only influence binding affinity but also modulate functional antagonism between miRNAs. These insights expand our understanding of miRNA specificity and underscore the need to consider 3′ end contributions when interpreting miRNA target networks in mammalian cells.

## Supplementary Material

gkag767_Supplemental_Files

## Data Availability

The mRNA-Seq and smallRNA-Seq data obtained after various miRNA and anti-miR transfections from HEK293T cells are publicly available at GEO (https://www.ncbi.nlm.nih.gov/geo/) under the accession code: GSE303559. The PCR-enriched chimeric eCLIP data (Manakov *et al*. [48]) analyzed in this study are available at GEO (accession code: GSE198250), and the original miR-CLIP data (Imig *et al*. [33]) are available at GEO (accession code: GSE62681). The original data used in this publication are available in a curated data archive at ETH Zurich (https://www.research-collection.ethz.ch) under the DOI 10.3929/ethz-c-000802621.
