# Supplementary material for: Seedless 3′ pairing enables miR-17 family miRNAs to seize let-7 target sites
Source: Nucleic Acids Res. 2026 Aug 14;54(15):gkag767. doi: 10.1093/nar/gkag767 (PMC13473885; doi:10.1093/nar/gkag767)
Supplement: Supplementary file 1 [file gkag767_supplemental_files.zip › Laski_NAR_SI-Resubmission.pdf]

Supplementary Information

Seedless 3′ pairing enables miR-17 family miRNAs to seize let-7 target sites

Artur Laski[1†*], Anneke Brümmer[2†], Matije Lucic[1†], Elisa Federici[3], Iris Mestres-Pascual[4], Jochen Imig[5], Alexander Kanitz[4], Mihaela Zavolan[4], Luca F. R. Gebert[6], Ian J. MacRae[6], Carlo V. Catapano[3] and Jonathan Hall[1*]

[1]Institute of Pharmaceutical Sciences, Department of Chemistry and Applied Biosciences, ETH Zurich; Zurich, Switzerland;

[†]These authors contributed equally.
*Correspondence: artur.laski@pharma.ethz.ch, jonathan.hall@pharma.ethz.ch.

Table of Contents

Table of Contents 1

Figure S1. *Percentage of canonical miR-106a target genes with 6mers complementary to the miR-106a sequence and fold changes of these genes after miR-106a transfection in HeLa cells.* 2

Figure S2. *MiR-106a counteracts let-7a-mediated suppression of LIN28B protein.* 3

Figure S3*. Chimeric eCLIP detects overlapping miR-17 and let-7a reads at predicted let-7a target sites in the HMGA2 3' UTR.* 4

Figure S4. *Binding of Ago2-miRNA complexes to let-7 target sites in HMGA2 and DICER1.* 5

Figure S5. *PAE plots of the AlphaFold3 predictions in Figures 4D and 4E.* 6

Figure S6. *Transcriptome-wide analysis of miR-106a and let-7a interaction in regulating let-7 target gene expression.* 7

Figure S7. *AntimiR-mediated inhibition of endogenous miRNA activity.* 8

Figure S8. *Alignment and expression profiles of let-7a, miR-106a, miR-106b, and miR-106a-5p mutants transfected into HEK293T cells.* 9

Figure S9. *PhastCons conservation scores around Targetscan-predicted miRNA target sites.* 10

Figure S10. *Abundance of miRNA 6mer and 7mer seeds in 3' end sequences of other miRNAs.* 11

Supplementary Tables 13

Supplementary Table S1*: Characterization of synthesized oligoribonucleotides.* 13

Supplementary Table S2*: qPCR primers used for SYBR green qPCR.* 14

Supplementary Table S3*: Luciferase plasmid reporter inserts.* 14

Supplementary Table S4*: Antibodies used for Western blotting.* 14

Supplementary Table S5*: Genes based on eCLIP peaks and log2 fold changes in miRNA transfection experiments.* 15

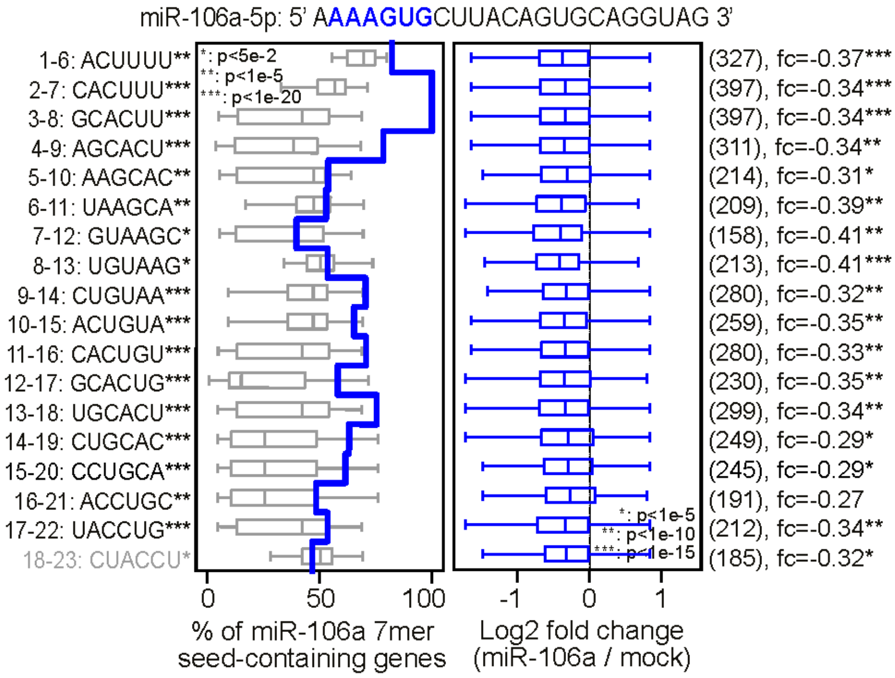

Figure S1. Percentage of canonical miR-106a target genes with 6mers complementary to the miR-106a sequence and fold changes of these genes after miR-106a transfection in HeLa cells.

Left panel: Blue line: Percentage of 397 mRNAs with miR-106a 7mer seeds (dark and medium blue groups in Figure 1C) that have a reverse complement 6mer match to the miR-106a sequence (indicated on the y-axis) in their 3′ UTRs; grey boxes: percentages of mRNAs with miR-106a 7mer seed that have matches to 6mers with shuffled nucleotides. P values are calculated using a one-sample t-test comparing the percentage of genes with miR-106a 6mer matches (blue line) to the percentages of genes with shuffled 6mer matches (grey boxes). Right panel: Boxplots of log2 fold changes after miR-106a transfection in HeLa cells for mRNAs with 6mer matches to the miR-106a sequence (indicated on the y-axis, left panel). The number of genes with 6mer matches, their median fold change, and p values are indicated on the right. P values are calculated using Wilcoxon rank-sum test to compare fold changes of each group of genes with those of all genes.

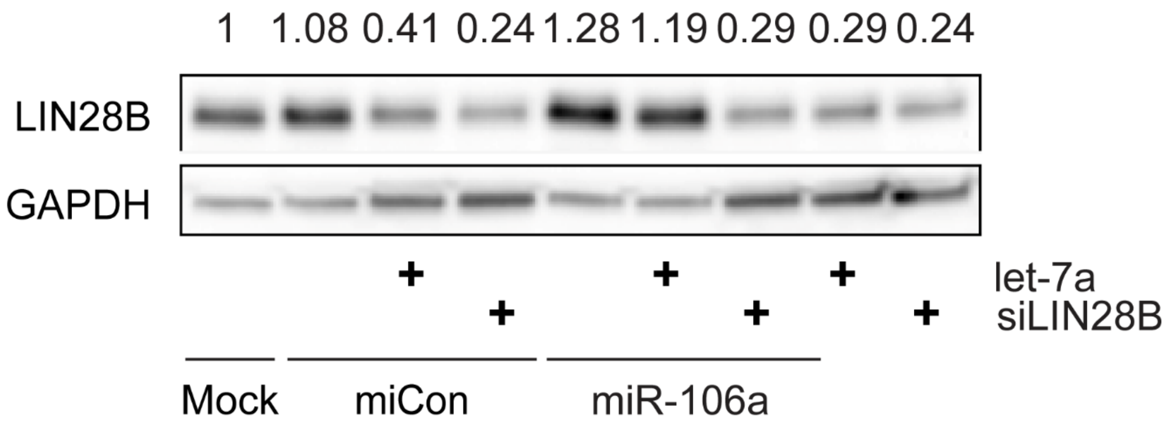

Figure S2. miR-106a counteracts let-7a-mediated suppression of LIN28B protein.

Western blot analysis of the validated let-7 target protein LIN28B and housekeeping control GAPDH in HEK293T cells transfected with 50 nM randomized negative control duplex (miCon) or miR-106a. The let-7a duplex and siLIN28B (positive knockdown control) were transfected at 10 nM either alone or in combination with negative control or miR-106a. Band intensities were normalized to the mock transfection (first lane) and the corresponding GAPDH signal, with normalized values indicated above the blots. A replicate western blot performed under conditions identical to those in Figure 3B is shown. These data support the results presented in Figure 3B.

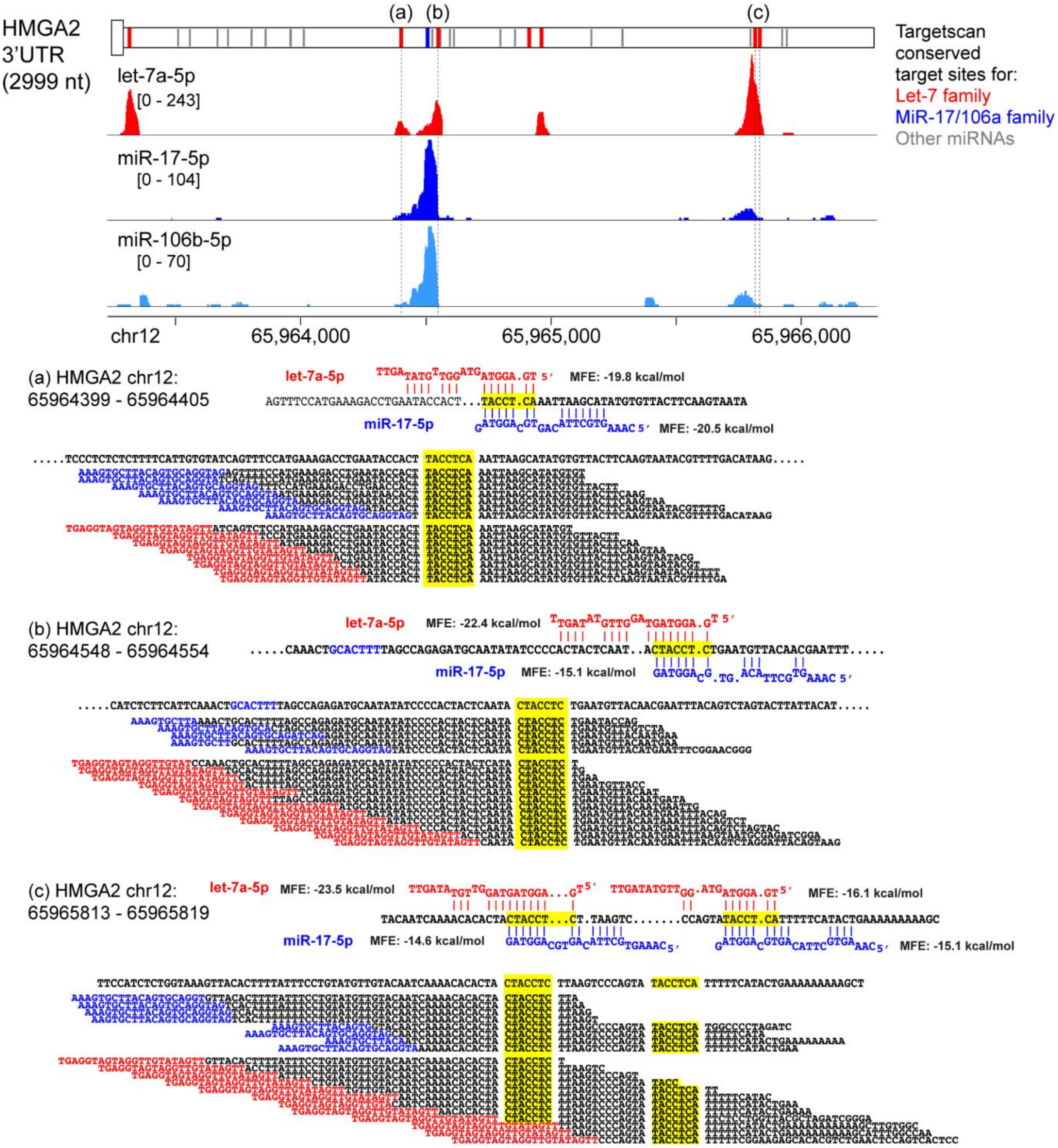

Figure S3. Chimeric eCLIP detects overlapping miR-17 and let-7a reads at predicted let-7a target sites in the HMGA2 3′ UTR.

Top panel: Genome browser view of chimeric eCLIP read densities across the HMGA2 3′ UTR for let-7a-5p (red), miR-17-5p (blue), and miR-106b-5p (light blue). Maximum read densities are indicated on the left for each miRNA track. Predicted miRNA target sites from TargetScan are shown as vertical lines for the let-7 family (red), miR-17/106a family (blue), and other miRNAs (grey) at top. Three regions containing predicted let-7 target sites with overlapping let-7a and miR-17 eCLIP reads are marked (a-c). **Panels (a-c):** Expanded views of the three marked regions. For each region, predicted base-pairing interactions determined by RNAhybrid are shown for let-7a (red) and miR-17 (blue). Below these predictions, representative raw chimeric eCLIP read pile-ups are shown (5-12 reads per site), with miR-17 reads in the upper group and let-7a reads in the lower group. The miRNA-derived sequence within each chimera is colored blue (miR-17) or red (let-7a), and the predicted target-site sequence within each read is highlighted in yellow. Notably, the first nucleotide is missing from all original miR-17 chimeric reads for an unknown reason, rendering the miRNA-derived sequence indistinguishable from miR-106a.

A HMGA2 let-7 target site at chr12: 65964548 - 65964554

GATGGTTGACCAAGGTGCTTTTCTTCGGCTTGAGTTCACCATCTCTTCATTCAAACTGCACTTTTAGCCAGAGATGCAATATATCCCCACTACTCAATACTACCTC

TGAATGTTACAACGAATTTACAGTCTAGTACTTATTACATGCTGCTATACACAAGCAATGCAAGAAAAAAAACTTACTGGGTAGGTGATTCTAATCATCTG

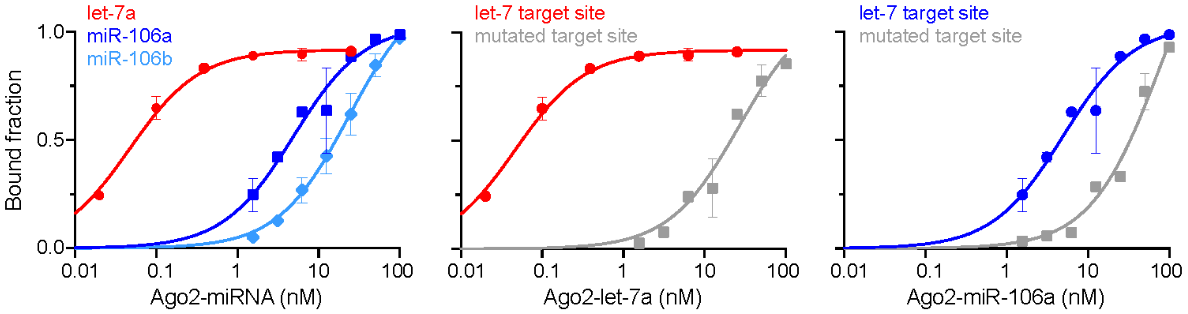

B DICER1 let-7 target site at chr14: 95087574 - 95087580

AGCTCATTATTTCCATCTTTGGAAAAGGAAAAAAAAAAAAAACTTCTTTAGGCATTTGCCTAAGTTTCTTTAATTAGACTTGTAGGCACTCTTCACTTAAATACCTC

AGTTCTTCTTTTCTTTTGCATGCATTTTTCCCCTGTTTGGTGCTATGTTTATGTATTATGCTTGAAATTTTAATTTTTTTTTTTTTGCACTGTAACTATAA

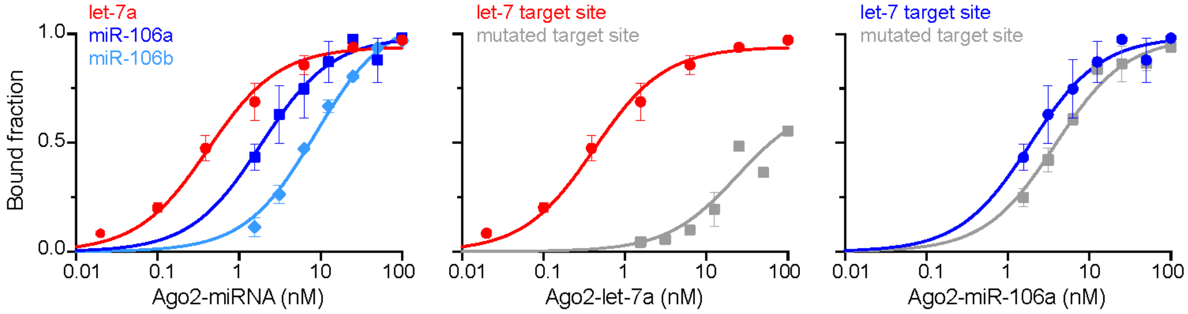

Figure S4. Binding of Ago2-miRNA complexes to let-7 target sites in HMGA2 and DICER1.

Bound fraction of let-7 target sites as a function of Ago2-miRNA concentration for a let-7 target site in (A) HMGA2 and (B) DICER1. (A,B) Top: genomic coordinates of, and nucleotide sequence around, the let-7a seed-complementary site (high-lighted with yellow). Left panels: Ago2 loaded with let-7a (red), miR-106a (blue), or miR-106b (cyan). Middle panels: Ago2-let-7a binding to wild-type target sites (red) and mutant target sites (grey), in which all nucleotides complementary to the let-7 seed were replaced by adenosines. Right panels: Ago2-miR-106a binding to wild-type target sites (blue) and mutant target sites (grey).

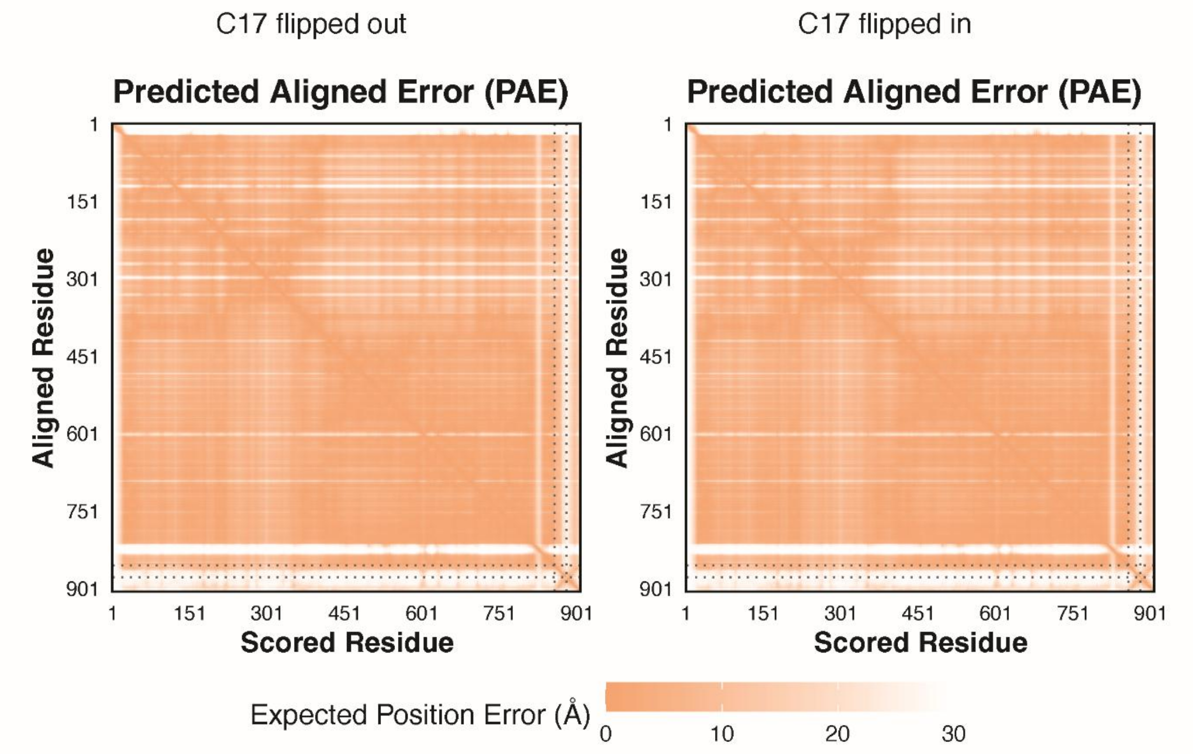

Figure S5. PAE plots of the AlphaFold3 predictions in Figures 4E and 4F.

Left panel, for C17 flipped out as demonstrated in 4E. Right panel for C17 flipped in as demonstrated in 4F. AGO2: residues 1-859; miR-106a: residues 860-882; HGMA2: residues 883-910).

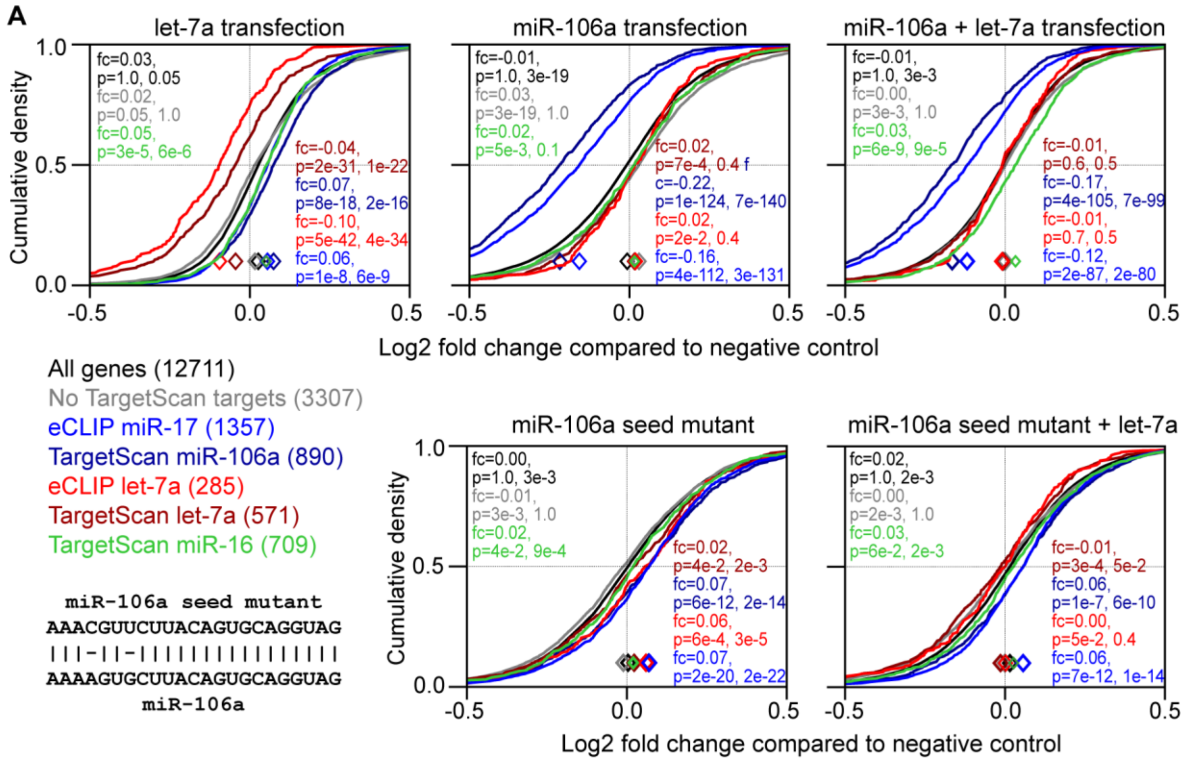

Figure S6. Transcriptome-wide analysis of miR-106a and let-7a interaction in regulating let-7 target gene expression.

Cumulative density function (CDF) plots of log2 fold changes in gene expression in HEK cells following different miRNA transfections, compared to a negative control. The top row panels show: left – let-7a transfection; middle – miR-106a transfection; right – co-transfection of let-7a and miR-106a. Each CDF curve represents one of seven gene groups: all genes (black), genes without any predicted miRNA target sites by TargetScan (grey), genes with eCLIP peaks for miR-17 in the 3′ UTR but not for let-7a (blue), genes with TargetScan-predicted sites for the miR-17 family but not for the let-7 family (dark blue), genes with eCLIP peaks for let-7a in the 3′ UTR but not for miR-17 (red), genes with TargetScan-predicted sites for the let-7 family but not for the miR-17 family (dark red), and genes with TargetScan-predicted sites for the miR-16 family and not for the let-7 or miR-17 families (green). Median fold changes for each group are indicated by diamonds. Each panel displays two p-values: the first compares each group to all genes; the second to genes without predicted target sites. The bottom row panels show: left – transfection with a miR-106a seed mutant containing two nucleotide substitutions at positions 4 and 7 (alignment shown); right – co-transfection of the seed mutant with let-7a. CDF curves represent the same gene groups as in the top row.

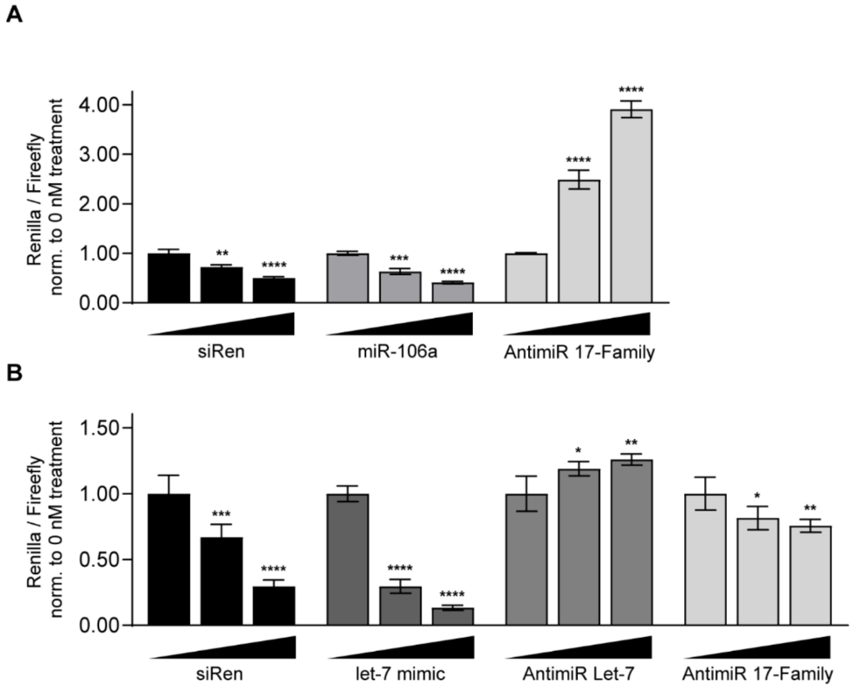

Figure S7. AntimiR-mediated inhibition of endogenous miRNA activity.

Relative luminescence of a fully complementary miR-106a sensor reporter (A) or a reporter containing the isolated HMGA2 3′UTR target site (Target 6) (B) in HEK293T cells. Cells were transfected with increasing concentrations (0, 2.5, 10, or 40 nM) of siRenilla (positive control), let-7a mimic, miR-106a mimic, let-7a antimiR, or a mixture of miR-17 family antimiRs (equimolar antimiR-17, -20, -93, and -106a). Renilla luminescence was normalized to Firefly and expressed relative to mock transfection (n = 3, set to 100%). Statistical significance was determined by two-way ANOVA with Dunnett's multiple comparisons against the 0 nM condition. Error bars represent SD. ns, not significant (omitted). *P ≤ 0.05; **P ≤ 0.01; ***P ≤ 0.001; ****P ≤ 0.0001.

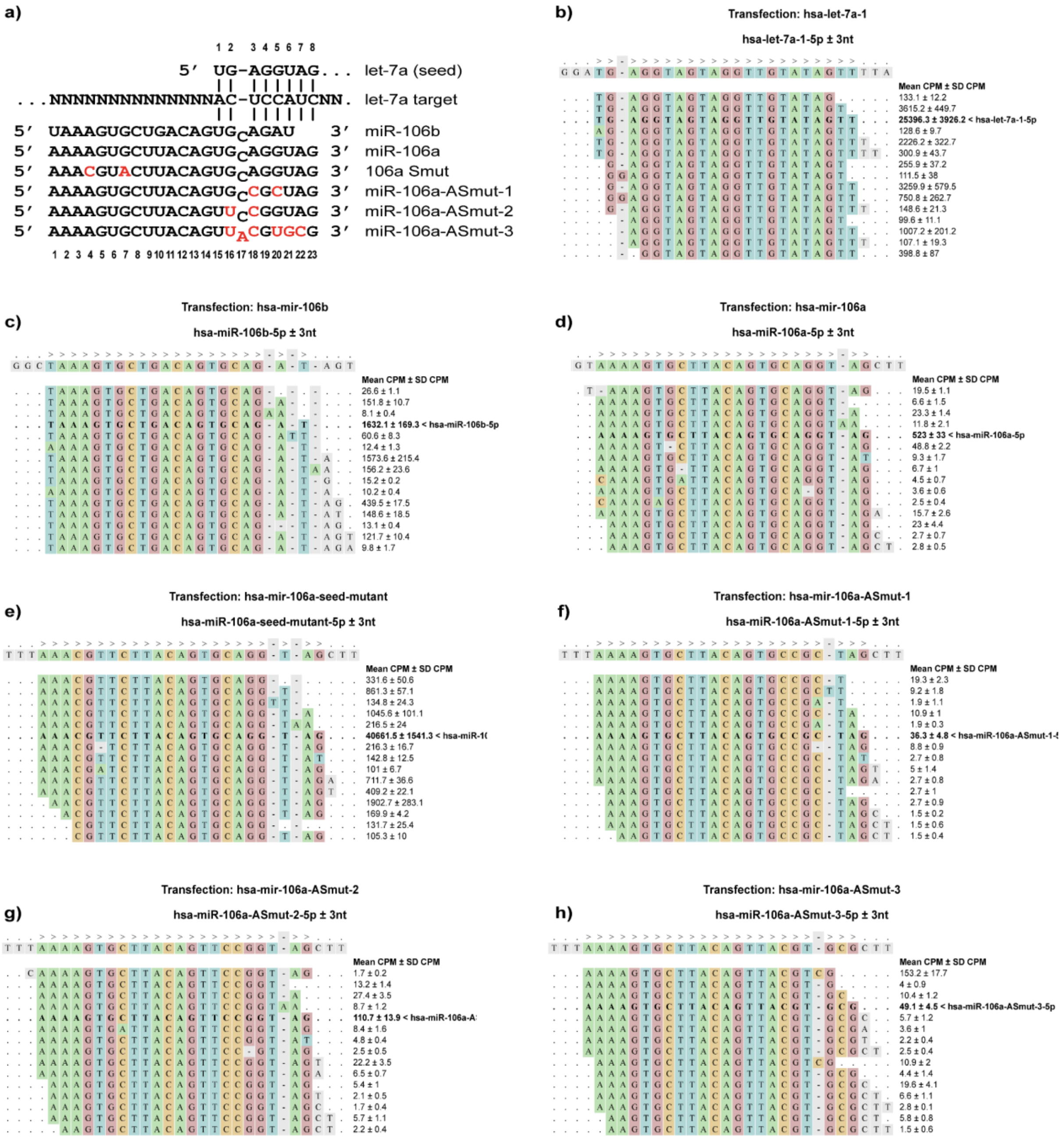

Figure S8. Alignment and expression profiles of let-7a, miR-106a, miR-106b, and miR-106a-5p mutants transfected into HEK293T cells.

Panel (**a**) shows the alignment of let-7a, miR-106a, miR-106b, and miR-106a-5p mutants to a canonical let-7 target site, with deviations relative to miR-106a-5p wild-type marked in red. Panels (b–h) show pileups of small RNA-Seq reads, processed as described in the Methods section from libraries extracted from HEK 293T cells transfected with 50 nM of: (**b**) pre-let-7a, (**c**) pre-miR-106b, (**d**) pre-miR-106a, (**e**) pre-miR-106a seed mutant, (**f**) pre-miR-106a anti-seed mutant-1, (**g**) pre-miR-106a anti-seed mutant 2, and (**h**) pre-miR-106a anti-seed mutant 3. Titles above panels b–h indicate the transfected construct (top) and the reference sequence (bottom) used for the alignments. For each pileup, the 15 most prevalent small RNA-Seq read species aligning to the 5p sequence of the transfected construct, ±3nt to account for processing variability, are indicated. The canonical 5p sequence is marked in bold. Indicated counts represent counts per million reads (CPM) and represent the means and standard deviations of three replicates.

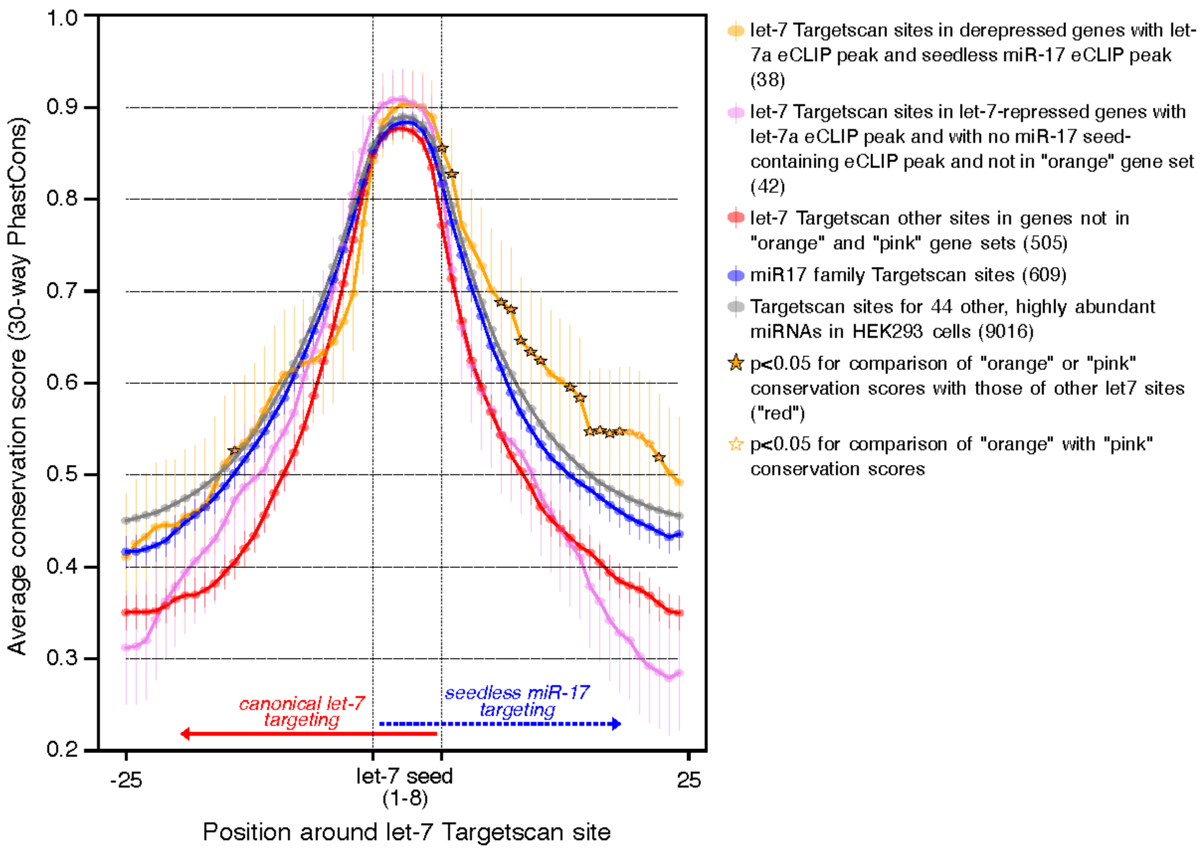

Figure S9. PhastCons conservation scores around Targetscan-predicted miRNA target sites.

PhastCons conservation scores within 25 nucleotides from Targetscan-predicted miRNA target sites for 5 groups of sites: predicted let-7 family target sites in genes that were derepressed in HEK293 cells (log2 fold change < -0.1 after let-7a transfection and log2 fold change >0 after let-7a + miR-106a double transfection compared to let-7a single transfection) and with a let-7a eCLIP peak and a seedless miR-17 eCLIP peak (no 6mer seed match between nucleotides 1-8) in their 3’UTR (shown in orange); predicted let-7 family target sites, not in the orange group, in genes that were repressed in HEK293 cells (log2 fold change < -0.1 after let-7a transfection) and had a let-7a eCLIP peak and no miR-17 6mer seed-containing eCLIP peak in their 3’UTR (violet); predicted let-7 family target sites, not in the orange and violet groups (red); predicted miR-17 family target sites (blue); and predicted target sites of other miRNA families with miRNAs among the top 50 expressed in HEK293 cells (gray). Thick lines with circles represent the average PhastCons scores for each group and position, and vertical lines indicate the standard error of the mean. Orange-filled stars indicate a significant difference between the PhastCons scores for the orange and red groups of predicted let-7 target sites at a specific position, while white-filled stars indicate a significant difference between the PhastCons scores for the orange and violet groups of predicted let-7 target sites (p value <0.05, Wilcoxon rank-sum test). Conservation scores were centered on TargetScan-predicted target sites, which define the precise seed-match position and thereby increase sensitivity for detecting differences in conservation at surrounding nucleotides. By contrast, centering on eCLIP peaks, which represent broader miRNA-bound regions without a precise targeting position, would dilute such positional differences. The orientation of the let-7 miRNA (from 5’ to 3’ end) engaged in canonical targeting is indicated by a red arrow at bottom, as well as the orientation of miR-17 (from 3’ to 5’ end) potentially engaged in noncanonical 3’end-mediated target interaction by a blue arrow.

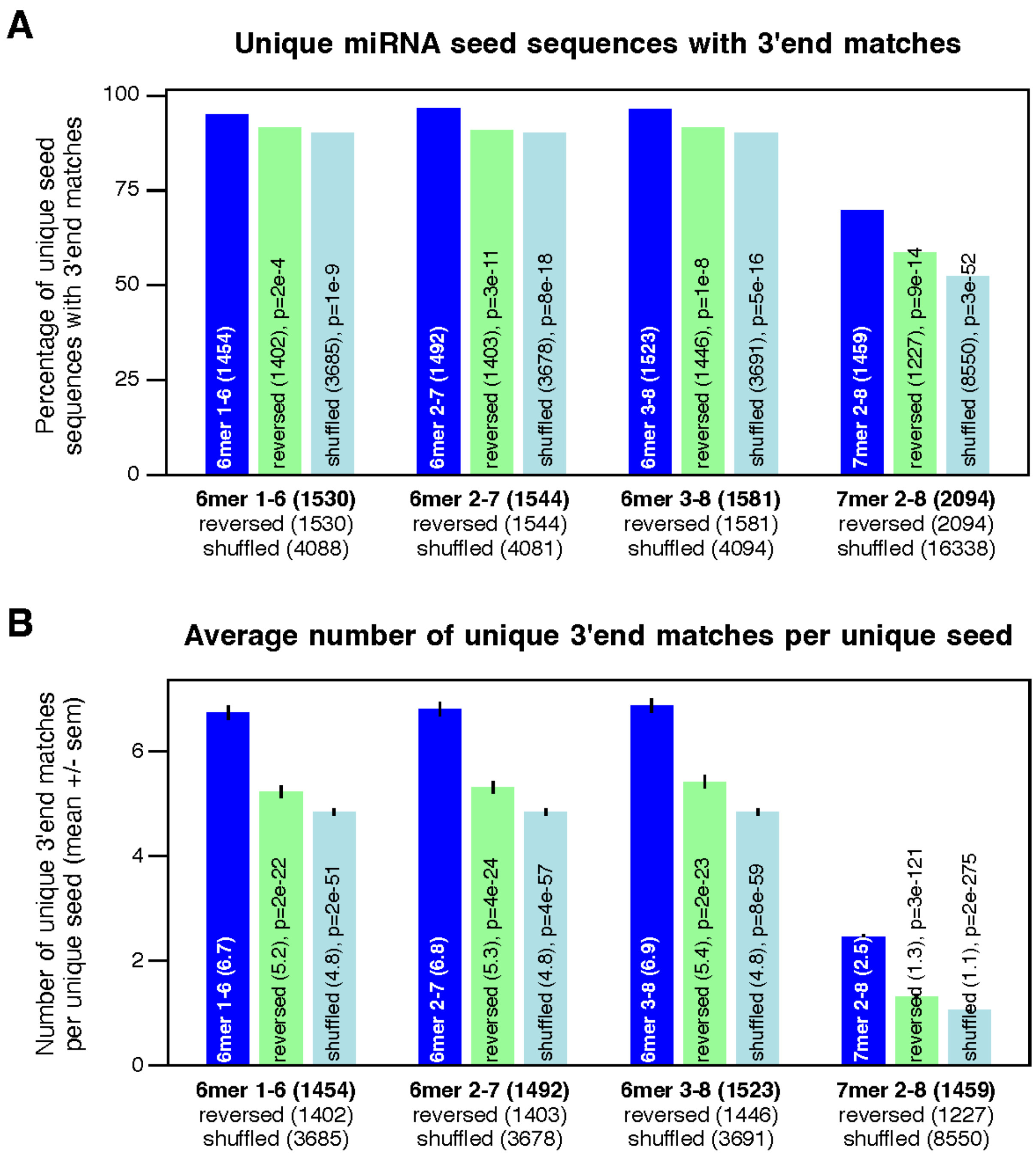

Figure S10. Abundance of miRNA 6mer and 7mer seeds in 3' end sequences of other miRNAs.

Unique 6mer and 7mer miRNA seed sequences with matches in 3' end sequences (starting from position 10) of other miRNAs (dark blue bars from left to right for: 6mers from position 1 to 6, 6mers from 2 to 7, 6mers from 3 to 8, and 7mers from 2 to 8). As a comparison, 3' end matches are analyzed for reverse 6mer and 7mer seed sequences (light green) and for shuffled 6mer and 7mer seed sequences (light blue). **(A)** Percentages of unique 6mer or 7mer sequences with at least one match in the 3' end sequence of another miRNA. P values are calculated using Fisher's exact test to compare the proportions with 3'end matches between reversed or shuffled and real miRNA 6mer/7mer seed sequences. P values are indicated inside reversed and shuffled bars. Numbers in brackets in each bar indicate the number of unique 6mers/7mers with 3'end matches. **(B)** Average number of 3'end matches for each unique 6mer/7mer sequence. Error bars indicate the standard error. P values are calculated using Wilcoxon's

ranksum test to compare the number of 3'end matches between reversed or shuffled and real miRNA 6mer/7mer seed sequences. P values are indicated inside reversed and shuffled bars. Numbers in brackets in each bar indicate the average number of 3'end matches per unique 6mer/7mer with 3'end match.

Supplementary Tables

Supplementary Table S1: Characterization of synthesized oligoribonucleotides.

NAME	SEQUENCE 5'→ 3'
**LET-7A DUPLEX**	5p-Strand: UGAGGUAGUAGGUUGUAUAGUU<br>3p-Strand: CUAUACAAUCUACUGUCUUUC
**MIR-106A**	AAAAGUGCUUACAGUGCAGGUAGCUUUUUGAGAUCUACUGCAAUGUA AGCACUUCUUAC
**MIR-106B**	UAAAGUGCUGACAGUGCAGAUAGUGGUCCUCUCCGUGCUACCGCACUG UGGGUACUUGCUGC
**106A SMUT**	AAACGUUCUUACAGUGCAGGUAGCUUUUUGAGAUCUACUGCAAUGUA AGAACGUCUUAC
**106A ASMUT-1**	AAAAGUGCUUACAGUGCCGCUAGCUUUUUGAGAUCUACGGCAAUGUA AGCACUUCUUAC
**106A ASMUT-2**	AAAAGUGCUUACAGUUCCGGUAGCUUUUUGAGAUCUACGGAAAUGUA AGCACUUCUUAC
**106A ASMUT-3**	AAAAGUGCUUACAGUUACGUGCGCUUUUUGAGAUCGCCGUAAAUGUA AGCACUUCUUAC
**SIRENILLA**	Guide Strand: GAGCGAAGAGGGCGAGAAAUU<br>Passenger Strand: AAUUUCUCGCCCUCUUCGCUC
**SILIN28B**	Guide Strand: AAAUCCUUCCAUGAAUAGUTT<br>Passenger Strand: ACUAUUCAUGGAAGGAUUUTT
**MIR-106A GUIDE**	5' phosphate AAAAGUGCUUACAGUGCAGGUAG
**MIR-106B GUIDE**	5' phosphate UAAAGUGCUGACAGUGCAGAU
**MIR-106 CAPTURE T1A**	biotin mUmCmUmAmGmUmGmUmAmGmCmAmCmUmAmGmCmCmGmC mAmCmUmUmUmAmAmCmUmCmU
**MIR-106 COMPETITOR FOR T1A**	biotin AGAGT**T**AAAGTGCGGCTAGTGCTACACTAGA
**LET-7A GUIDE**	5' phosphate UGAGGUAGUAGGUUGUAUAGUU
**LET-7A CAPTURE**	mAmUmAmGmAmCmUmGmCmGmAmCmAmAmUmAmGmCmCmUm AmCmCmUmCmCmGmAmAmCmG
**LET-7A COMPETITOR**	biotin CGTTCGGAGGTAGGCTATTGTCGCAGTCTAT
**T7 PROMOTER**	TTCTAATACGACTCACTATAG
**HMGA SITE 2 TEMPLATE**	ACTAGACTGTAAATTCGTTGTAACATTCA**GAGGTA**GTATTGAGTAGTGG GGATATATTGCATCTCCCTATAGTGAGTCGTATTAGAA
**HMGA SITE 2 POLYA CONTROL TEMPLATE**	ACTAGACTGTAAATTCGTTGTAACATTCA**TTTTTT**GTATTGAGTAGTGGG GATATATTGCATCTCCCTATAGTGAGTCGTATTAGAA
**DICER SITE 1 TEMPLATE**	GAAAAATGCATGCAAAAGAAAAGAAGAACT**GAGGTA**TTTAAGTGAAGA GTGCCTACAAGTCCCTATAGTGAGTCGTATTAGAA
**DICER SITE 1 POLYA CONTROL TEMPLATE**	GAAAAATGCATGCAAAAGAAAAGAAGAACT**TTTTTT**TTTAAGTGAAGA GTGCCTACAAGTCCCTATAGTGAGTCGTATTAGAA

Supplementary Table S2: qPCR primers used for SYBR green qPCR.

GENE	FORWARD PRIMER	REVERSE PRIMER
**ACTB**	CCAACCGCGAGAAGATGA	CCAGAGGCGTACAGGGATAG
**ANKRD52**	GGACGAGCCACTGAAGGAG	GGTCTGCACCGTTATCCAGT
**ARID3B**	GATGGCACCACCTATGCAG	GAGCAGACCCCGTGATGA
**DICER1**	GTCCGATGGTTCTCGAAGG	GCAAAGCAGGGCTTTTCA
**GAPDH**	AGCCACATCGCTCAGACAC	GCCCAATACGACCAAATCC
**HMGA2**	TCCCTCTAAAGCAGCTCAAAA	ACTTGTTGTGGCCATTTCCT
**LIN28B**	GAAAAGAAAACCAAAGGGAGATAGA	GAGGTAGACTACATTCCTTAGCATGA

Supplementary Table S3: Luciferase plasmid reporter inserts.

NAME	BACKBONE	INSERT SEQUENCE
**HMGA2 3′ UTR**	psiCHECK-2 Promega #C8021	HMGA2 3′ UTR (cloned from Addgene plasmid #14785).
**ANKRD52 MiR17TS**	psiCHECK-2 Promega #C8021	TTTAGAAAAAGTCTAAACATTTAGGGCACTTTAAAGGAGACACTC CTAGCCTGGCCCCTAACCACGCACTTTAACCTTGCCTAAAGCACT TGCTTCAAGTAATAAGCACTTTTGTGAAAA

Supplementary Table S4: Antibodies used for Western blotting.

NAME	CATALOGUE NR.
**anti-*GAPDH***	CST, #4196S
**anti-*LIN28B***	Origene, #TA802519

Supplementary Table S5: Selected genes based on eCLIP peaks and log2 fold changes in miRNA transfection experiments in HEK293T cells.

Columns 6-9 indicate the largest overlap (in nucleotides) between eCLIP peaks for let-7 (family) and miR-17 (family).

GeneID	Gene name	log2FC let7a / neg	log2FC (let7a + miR106a) / neg	Delta: log2FC (let7a + miR106a) / let7a	Overlap between let-7a and miR-17 eCLIP peaks (nt)	Overlap between let-7a and seedless miR-17 eCLIP peaks (nt)	Overlap between let-7 family (a,e,f,g) and miR-17 family (17,20a,93) eCLIP peaks (nt)	Overlap between let-7 family (a,e,f,g) and seedless miR-17 family (17,20a,93) eCLIP peaks (nt)
ENSG00000206557	TRIM71	-1.004	0.226	1.229	56	56	63	63
ENSG00000149948	HMGA2	-1.678	-0.494	1.184	66	40	66	42
ENSG00000187772	LIN28B	-1.256	-0.179	1.077	0	0	0	0
ENSG00000165655	ZNF503	-0.143	0.783	0.926	0	0	0	0
ENSG00000174498	IGDCC3	-1.378	-0.602	0.777	22	0	30	0
ENSG00000113196	HAND1	-0.884	-0.128	0.756	64	64	64	64
ENSG00000078399	HOXA9	-0.241	0.490	0.731	43	43	50	50
ENSG00000148200	NR6A1	-0.646	0.081	0.727	0	0	8	8
ENSG00000100697	DICER1	-0.808	-0.139	0.669	42	42	44	44
ENSG00000114739	ACVR2B	-0.508	0.150	0.658	0	0	0	0
ENSG00000166450	PRTG	-0.724	-0.071	0.653	0	0	0	0
ENSG00000146109	ABT1	-0.624	-0.045	0.579	0	0	0	0
ENSG00000185950	IRS2	-0.149	0.395	0.543	0	0	0	0
ENSG00000159217	IGF2BP1	-0.961	-0.433	0.529	50	50	54	50
ENSG00000155189	AGPAT5	-0.191	0.332	0.523	0	0	0	0
ENSG00000160131	VMA21	-0.061	0.461	0.522	50	50	82	82
ENSG00000146083	RNF44	-0.280	0.227	0.507	50	50	50	50
ENSG00000136603	SKIL	-0.404	0.099	0.502	0	0	48	0
ENSG00000167470	MIDN	-0.258	0.235	0.493	0	0	0	0
ENSG00000140450	ARRDC4	-0.289	0.200	0.489	19	19	21	19
ENSG00000181450	ZNF678	-0.127	0.345	0.472	0	0	25	0
ENSG00000106004	HOXA5	-0.058	0.403	0.461	12	12	25	25
ENSG00000123728	RAP2C	-0.038	0.420	0.458	54	54	54	54
ENSG00000165389	SPTSSA	-0.118	0.326	0.444	0	0	0	0
ENSG00000100528	CNIH1	-0.129	0.311	0.440	24	0	28	0
ENSG00000174021	GNG5	-0.454	-0.020	0.434	46	46	50	50
ENSG00000165434	PGM2L1	-0.461	-0.035	0.426	50	0	50	0
ENSG00000173674	EIF1AX	-0.132	0.294	0.426	0	0	0	0
ENSG00000183779	ZNF703	-0.013	0.398	0.410	49	49	49	49
ENSG00000129128	SPCS3	-0.063	0.346	0.409	0	0	0	0
ENSG00000167173	C15orf39	-0.342	0.063	0.405	0	0	0	0
ENSG00000049245	VAMP3	-0.378	0.022	0.401	46	0	60	0
ENSG00000143742	SRP9	-0.136	0.249	0.385	50	50	50	50
ENSG00000137996	RTCA	-0.391	-0.007	0.384	0	0	47	47

ENSG00000164683	HEY1	-0.242	0.140	0.382	0	0	0	0
ENSG00000158156	XKR8	-0.295	0.085	0.381	0	0	0	0
ENSG00000086712	TXLNG	-0.180	0.197	0.376	0	0	49	49
ENSG00000087502	ERGIC2	-0.262	0.106	0.368	1	0	46	0
ENSG00000062716	VMP1	-0.156	0.201	0.357	48	48	49	49
ENSG00000157224	CLDN12	-0.256	0.101	0.357	0	0	0	0
ENSG00000067533	RRP15	-0.315	0.040	0.355	0	0	0	0
ENSG00000108523	RNF167	-0.056	0.298	0.354	53	53	53	53
ENSG00000160208	RRP1B	-0.116	0.237	0.352	0	0	0	0
ENSG00000254870	ATP6V1G2<br>-DDX39B	-0.346	0.003	0.349	42	36	59	48
ENSG00000115234	SNX17	-0.132	0.213	0.344	0	0	0	0
ENSG00000105997	HOXA3	-0.145	0.192	0.338	11	11	17	17
ENSG00000124783	SSR1	-0.080	0.252	0.332	49	49	49	49
ENSG00000135535	CD164	-0.245	0.084	0.329	0	0	49	49
ENSG00000163376	KBTBD8	-0.366	-0.040	0.325	49	49	49	49
ENSG00000102081	FMR1	-0.056	0.266	0.322	0	0	48	48
ENSG00000139800	ZIC5	-0.057	0.264	0.320	0	0	0	0
ENSG00000141429	GALNT1	-0.132	0.183	0.315	0	0	0	0
ENSG00000165997	ARL5B	-0.104	0.211	0.314	0	0	0	0
ENSG00000179387	ELMOD2	-0.135	0.175	0.310	0	0	57	0
ENSG00000120519	SLC10A7	-0.382	-0.073	0.309	1	1	11	11
ENSG00000174840	PDE12	-0.226	0.082	0.307	0	0	0	0
ENSG00000139263	LRIG3	-0.423	-0.116	0.307	50	0	50	0
ENSG00000126067	PSMB2	-0.311	-0.006	0.305	0	0	0	0
ENSG00000111276	CDKN1B	-0.142	0.161	0.302	39	39	62	62
ENSG00000174695	TMEM167<br>A	-0.324	-0.023	0.301	0	0	0	0
ENSG00000114302	PRKAR2A	-0.221	0.070	0.291	0	0	0	0
ENSG00000130766	SESN2	-0.248	0.042	0.290	49	49	50	50
ENSG00000164134	NAA15	-0.189	0.100	0.289	0	0	0	0
ENSG00000142864	SERBP1	-0.052	0.234	0.287	0	0	40	40
ENSG00000124532	MRS2	-0.348	-0.062	0.285	0	0	0	0
ENSG00000103342	GSPT1	-0.143	0.138	0.281	50	50	50	50
ENSG00000154305	MIA3	-0.026	0.253	0.279	53	53	87	87
ENSG00000138593	SECISBP2<br>L	-0.076	0.202	0.278	32	32	32	32
ENSG00000107443	CCNJ	-0.238	0.036	0.274	34	34	45	45
ENSG00000116747	RO60	-0.119	0.155	0.274	0	0	0	0
ENSG00000133193	FAM104A	-0.229	0.043	0.272	0	0	0	0
ENSG00000110987	BCL7A	-0.021	0.251	0.271	0	0	0	0
ENSG00000143641	GALNT2	-0.259	0.010	0.269	0	0	0	0
ENSG00000117505	DR1	-0.031	0.236	0.267	0	0	0	0
ENSG00000164466	SFXN1	-0.085	0.181	0.266	51	51	54	54
ENSG00000180901	KCTD2	-0.103	0.163	0.266	0	0	47	47
ENSG00000169946	ZFPM2	-0.240	0.022	0.262	0	0	0	0
ENSG00000107864	CPEB3	-0.134	0.126	0.260	0	0	4	4

ENSG00000037965	HOXC8	-0.273	-0.018	0.256	65	65	65	65
ENSG00000088247	KHSRP	-0.124	0.131	0.256	0	0	0	0
ENSG00000153250	RBMS1	-0.343	-0.088	0.255	0	0	0	0
ENSG00000170571	EMB	-0.089	0.165	0.254	0	0	0	0
ENSG00000153006	SREK1IP1	-0.224	0.028	0.252	0	0	0	0
ENSG00000141682	PMAIP1	-0.196	0.056	0.252	59	0	61	0
ENSG00000116604	MEF2D	-0.049	0.203	0.252	0	0	0	0
ENSG00000278053	DDX52	-0.142	0.108	0.250	0	0	0	0
ENSG00000143815	LBR	-0.121	0.128	0.248	17	17	19	19
ENSG00000065135	GNAI3	-0.126	0.122	0.247	49	49	50	50
ENSG00000052795	FNIP2	-0.304	-0.059	0.245	0	0	0	0
ENSG00000131732	ZCCHC9	-0.309	-0.068	0.241	50	50	62	62
ENSG00000092621	PHGDH	-0.010	0.230	0.241	0	0	0	0
ENSG00000177125	ZBTB34	-0.081	0.160	0.241	0	0	45	45
ENSG00000125772	GPCPD1	-0.356	-0.117	0.239	0	0	0	0
ENSG00000008394	MGST1	-0.204	0.033	0.237	47	0	47	0
ENSG00000241484	ARHGAP8	-0.319	-0.089	0.229	45	0	47	0
ENSG00000143674	MAP3K21	-0.020	0.209	0.228	0	0	0	0
ENSG00000069702	TGFBR3	-0.509	-0.281	0.228	0	0	0	0
ENSG00000173065	FAM222B	-0.419	-0.191	0.228	0	0	0	0
ENSG00000099194	SCD	-0.100	0.125	0.225	0	0	0	0
ENSG00000150907	FOXO1	-0.234	-0.009	0.225	0	0	0	0
ENSG00000277586	NEFL	-0.266	-0.041	0.225	50	50	50	50
ENSG00000123505	AMD1	-0.035	0.180	0.215	0	0	0	0
ENSG00000136381	IREB2	-0.044	0.171	0.215	44	44	50	50
ENSG00000085491	SLC25A24	-0.407	-0.192	0.215	0	0	0	0
ENSG00000160058	BSDC1	-0.104	0.110	0.214	39	31	50	45
ENSG00000046604	DSG2	-0.091	0.122	0.213	0	0	42	42
ENSG00000112679	DUSP22	-0.361	-0.149	0.212	0	0	0	0
ENSG00000239900	ADSL	-0.237	-0.027	0.210	52	0	52	0
ENSG00000125447	GGA3	-0.135	0.075	0.210	0	0	47	47
ENSG00000119042	SATB2	-0.164	0.039	0.204	50	0	50	0
ENSG00000174437	ATP2A2	-0.107	0.096	0.203	0	0	0	0
ENSG00000184009	ACTG1	-0.208	-0.006	0.202	42	42	42	42
ENSG00000121579	NAA50	-0.061	0.140	0.201	0	0	0	0
ENSG00000112081	SRSF3	-0.071	0.129	0.200	0	0	0	0
ENSG00000117500	TMED5	-0.151	0.047	0.198	49	49	50	50
ENSG00000152683	SLC30A6	-0.080	0.113	0.193	0	0	0	0
ENSG00000166986	MARS1	-0.109	0.084	0.193	0	0	0	0
ENSG00000114770	ABCC5	-0.269	-0.078	0.192	0	0	0	0
ENSG00000132640	BTBD3	-0.158	0.031	0.189	0	0	0	0
ENSG00000123472	ATPAF1	-0.211	-0.024	0.186	0	0	18	0
ENSG00000089248	ERP29	-0.215	-0.031	0.184	0	0	0	0
ENSG00000168291	PDHB	-0.241	-0.058	0.183	28	28	28	28

ENSG00000142252	GEMIN7	-0.439	-0.257	0.182	0	0	0	0
ENSG00000124613	ZNF391	-0.215	-0.035	0.180	0	0	0	0
ENSG00000120742	SERP1	-0.132	0.048	0.180	0	0	0	0
ENSG00000113758	DBN1	-0.086	0.092	0.178	0	0	0	0
ENSG00000144674	GOLGA4	-0.041	0.135	0.177	0	0	0	0
ENSG00000159445	THEM4	-0.136	0.039	0.175	0	0	0	0
ENSG00000126775	ATG14	-0.124	0.048	0.172	0	0	0	0
ENSG00000089006	SNX5	-0.089	0.081	0.170	0	0	0	0
ENSG00000165300	SLITRK5	-0.072	0.093	0.165	0	0	0	0
ENSG00000196911	KPNA5	-0.195	-0.031	0.164	0	0	53	53
ENSG00000124222	STX16	-0.051	0.110	0.161	0	0	0	0
ENSG00000167088	SNRPD1	-0.123	0.036	0.159	0	0	50	50
ENSG00000119787	ATL2	-0.062	0.097	0.159	67	67	68	68
ENSG00000137309	HMGA1	-0.152	0.006	0.158	0	0	0	0
ENSG00000082269	FAM135A	-0.138	0.019	0.157	0	0	0	0
ENSG00000103742	IGDCC4	-0.277	-0.120	0.157	44	44	48	48
ENSG00000160014	CALM3	-0.278	-0.122	0.156	0	0	0	0
ENSG00000172667	ZMAT3	-0.129	0.027	0.156	0	0	0	0
ENSG00000084090	STARD7	-0.054	0.100	0.155	0	0	0	0
ENSG00000148730	EIF4EBP2	-0.235	-0.080	0.154	49	49	49	49
ENSG00000172795	DCP2	-0.056	0.098	0.154	0	0	0	0
ENSG00000197930	ERO1A	-0.356	-0.202	0.154	50	0	76	0
ENSG00000110321	EIF4G2	-0.365	-0.211	0.154	0	0	50	0
ENSG00000118579	MED28	-0.227	-0.075	0.152	0	0	0	0
ENSG00000160679	CHTOP	-0.045	0.107	0.152	52	15	64	20
ENSG00000104290	FZD3	-0.197	-0.046	0.151	0	0	0	0
ENSG00000100077	GRK3	-0.170	-0.022	0.148	0	0	0	0
ENSG00000102931	ARL2BP	-0.067	0.080	0.147	0	0	0	0
ENSG00000153187	HNRNPU	-0.103	0.044	0.147	0	0	65	0
ENSG00000144231	POLR2D	-0.113	0.033	0.146	0	0	0	0
ENSG00000140950	MEAK7	-0.383	-0.237	0.146	23	0	23	0
ENSG00000057252	SOAT1	-0.120	0.026	0.146	0	0	0	0
ENSG00000149923	PPP4C	-0.204	-0.059	0.145	50	50	50	50
ENSG00000112218	GPR63	-0.111	0.033	0.145	0	0	0	0
ENSG00000100320	RBFOX2	-0.132	0.012	0.144	46	0	51	0
ENSG00000145685	LHFPL2	-0.044	0.100	0.144	0	0	0	0
ENSG00000164284	GRPEL2	-0.011	0.133	0.144	0	0	0	0
ENSG00000137710	RDX	-0.173	-0.031	0.142	0	0	0	0
ENSG00000084234	APLP2	-0.177	-0.038	0.138	0	0	0	0
ENSG00000128989	ARPP19	-0.120	0.017	0.137	0	0	0	0
ENSG00000164651	SP8	-0.505	-0.370	0.135	0	0	0	0
ENSG00000170242	USP47	-0.143	-0.009	0.134	0	0	0	0
ENSG00000158417	EIF5B	-0.163	-0.031	0.131	40	0	49	0
ENSG00000150593	PDCD4	-0.026	0.106	0.131	0	0	0	0

ENSG00000134419	RPS15A	-0.149	-0.018	0.131	0	0	0	0
ENSG00000204116	CHIC1	-0.276	-0.146	0.130	0	0	0	0
ENSG00000166483	WEE1	-0.043	0.087	0.130	26	0	27	0
ENSG00000187109	NAP1L1	-0.152	-0.024	0.129	0	0	0	0
ENSG00000182263	FIGN	-0.280	-0.153	0.128	50	50	50	50
ENSG00000118971	CCND2	-0.173	-0.046	0.127	0	0	49	49
ENSG00000167770	OTUB1	-0.115	0.012	0.127	0	0	48	48
ENSG00000064393	HIPK2	-0.022	0.104	0.126	0	0	0	0
ENSG00000095015	MAP3K1	-0.276	-0.150	0.126	0	0	43	0
ENSG00000112394	SLC16A10	-0.073	0.050	0.123	0	0	0	0
ENSG00000180758	GPR157	-0.302	-0.179	0.122	0	0	0	0
ENSG00000188811	NHLRC3	-0.102	0.019	0.121	0	0	50	50
ENSG00000134278	SPIRE1	-0.137	-0.016	0.121	0	0	0	0
ENSG00000100811	YY1	-0.086	0.034	0.120	0	0	0	0
ENSG00000116991	SIPA1L2	-0.086	0.032	0.118	9	0	19	0
ENSG00000122566	HNRNPA2B1	-0.075	0.043	0.118	0	0	0	0
ENSG00000162923	WDR26	-0.034	0.083	0.117	0	0	0	0
ENSG00000164933	SLC25A32	-0.097	0.020	0.117	0	0	0	0
ENSG00000005893	LAMP2	-0.123	-0.009	0.114	0	0	0	0
ENSG00000213160	KLHL23	-0.084	0.029	0.114	50	0	50	0
ENSG00000128590	DNAJB9	-0.173	-0.059	0.114	20	20	28	28
ENSG00000114861	FOXP1	-0.139	-0.026	0.113	0	0	0	0
ENSG00000006831	ADIPOR2	-0.082	0.030	0.112	0	0	0	0
ENSG00000172113	NME6	-0.209	-0.100	0.109	0	0	0	0
ENSG00000166803	PCLAF	-0.025	0.083	0.108	0	0	0	0
ENSG00000109184	DCUN1D4	-0.041	0.066	0.106	0	0	0	0
ENSG00000179476	C14orf28	-0.115	-0.009	0.106	26	0	29	0
ENSG00000114030	KPNA1	-0.140	-0.034	0.106	0	0	0	0
ENSG00000110917	MLEC	-0.012	0.094	0.106	42	42	47	47
ENSG00000146373	RNF217	-0.113	-0.011	0.102	0	0	21	0
ENSG00000141425	RPRD1A	-0.018	0.083	0.102	0	0	0	0
ENSG00000163684	RPP14	-0.115	-0.017	0.099	0	0	0	0
ENSG00000173320	STOX2	-0.068	0.029	0.097	0	0	0	0
ENSG00000171302	CANT1	-0.330	-0.234	0.096	0	0	0	0
ENSG00000110367	DDX6	-0.023	0.070	0.094	0	0	0	0
ENSG00000139734	DIAPH3	-0.059	0.034	0.093	9	9	9	9
ENSG00000145439	CBR4	-0.118	-0.025	0.093	0	0	0	0
ENSG00000196428	TSC22D2	-0.068	0.025	0.092	48	0	56	0
ENSG00000116731	PRDM2	-0.257	-0.165	0.092	2	0	12	0
ENSG00000241258	CRCP	-0.067	0.024	0.091	0	0	0	0
ENSG00000135912	TTLL4	-0.392	-0.301	0.091	0	0	21	0
ENSG00000185818	NAT8L	-0.055	0.036	0.091	0	0	0	0
ENSG00000162613	FUBP1	-0.099	-0.012	0.087	47	0	50	0
ENSG00000106086	PLEKHA8	-0.239	-0.153	0.085	0	0	0	0

ENSG00000065320	NTN1	-0.312	-0.227	0.085	0	0	0	0
ENSG00000187231	SESTD1	-0.012	0.073	0.085	0	0	0	0
ENSG00000116106	EPHA4	-0.338	-0.253	0.085	71	0	72	0
ENSG00000095739	BAMBI	-0.359	-0.276	0.083	8	0	8	0
ENSG00000145907	G3BP1	-0.162	-0.080	0.082	0	0	0	0
ENSG00000134987	WDR36	-0.051	0.029	0.081	7	7	12	12
ENSG00000124171	PARD6B	-0.271	-0.191	0.080	30	0	34	0
ENSG00000164167	LSM6	-0.230	-0.152	0.078	0	0	0	0
ENSG00000075624	ACTB	-0.037	0.040	0.077	0	0	49	49
ENSG00000065308	TRAM2	-0.047	0.029	0.077	0	0	0	0
ENSG00000197498	RPF2	-0.179	-0.107	0.073	55	0	57	0
ENSG00000121671	CRY2	-0.044	0.023	0.067	58	58	58	58
ENSG00000143384	MCL1	-0.012	0.054	0.066	0	0	62	0
ENSG00000197037	ZSCAN25	-0.177	-0.111	0.066	0	0	0	0
ENSG00000078403	MLLT10	-0.081	-0.015	0.066	0	0	0	0
ENSG00000137266	SLC22A23	-0.046	0.018	0.065	0	0	0	0
ENSG00000076067	RBMS2	-0.131	-0.067	0.065	0	0	0	0
ENSG00000116990	MYCL	-0.072	-0.010	0.062	55	0	57	0
ENSG00000167081	PBX3	-0.304	-0.242	0.062	0	0	0	0
ENSG00000108064	TFAM	-0.075	-0.013	0.061	50	0	50	0
ENSG00000140443	IGF1R	-0.406	-0.345	0.061	0	0	42	42
ENSG00000117713	ARID1A	-0.121	-0.060	0.061	0	0	0	0
ENSG00000261609	GAN	-0.072	-0.013	0.059	0	0	0	0
ENSG00000116141	MARK1	-0.024	0.033	0.057	0	0	0	0
ENSG00000221914	PPP2R2A	-0.077	-0.021	0.056	0	0	0	0
ENSG00000256235	SMIM3	-0.052	0.004	0.056	0	0	0	0
ENSG00000113732	ATP6V0E1	-0.185	-0.130	0.055	50	0	50	0
ENSG00000153201	RANBP2	-0.120	-0.065	0.054	0	0	0	0
ENSG00000087152	ATXN7L3	-0.044	0.009	0.053	0	0	0	0
ENSG00000161048	NAPEPLD	-0.095	-0.043	0.053	39	0	73	0
ENSG00000151233	GXYLT1	-0.065	-0.012	0.053	0	0	0	0
ENSG00000141568	FOXK2	-0.056	-0.004	0.052	0	0	0	0
ENSG00000166402	TUB	-0.018	0.033	0.051	0	0	0	0
ENSG00000164597	COG5	-0.108	-0.058	0.050	51	0	64	0
ENSG00000166233	ARIH1	-0.013	0.037	0.050	0	0	0	0
ENSG00000166398	GARRE1	-0.022	0.027	0.049	0	0	0	0
ENSG00000143190	POU2F1	-0.151	-0.102	0.049	0	0	0	0
ENSG00000100441	KHNYN	-0.114	-0.068	0.046	0	0	0	0
ENSG00000101596	SMCHD1	-0.029	0.016	0.044	44	44	47	47
ENSG00000139266	MARCHF9	-0.109	-0.065	0.044	0	0	24	24
ENSG00000103202	NME4	-0.177	-0.135	0.042	37	37	43	43
ENSG00000164631	ZNF12	-0.125	-0.084	0.041	0	0	0	0
ENSG00000122085	MTERF4	-0.055	-0.016	0.038	0	0	0	0
ENSG00000164045	CDC25A	-0.289	-0.251	0.038	0	0	0	0

ENSG00000215301	DDX3X	-0.037	-0.000	0.037	4	4	4	4
ENSG00000135503	ACVR1B	-0.203	-0.170	0.033	51	0	57	0
ENSG00000196182	STK40	-0.158	-0.126	0.033	0	0	0	0
ENSG00000168906	MAT2A	-0.030	-0.000	0.030	0	0	0	0
ENSG00000079819	EPB41L2	-0.068	-0.040	0.029	0	0	0	0
ENSG00000143401	ANP32E	-0.028	0.000	0.028	0	0	0	0
ENSG00000102024	PLS3	-0.107	-0.081	0.026	0	0	0	0
ENSG00000196632	WNK3	-0.163	-0.138	0.025	0	0	4	0
ENSG00000183814	LIN9	-0.081	-0.056	0.025	0	0	0	0
ENSG00000197006	METTL9	-0.007	0.018	0.025	0	0	0	0
ENSG00000171735	CAMTA1	-0.018	0.006	0.024	0	0	0	0
ENSG00000119689	DLST	-0.090	-0.065	0.024	0	0	0	0
ENSG00000135679	MDM2	-0.134	-0.111	0.023	0	0	0	0
ENSG00000181026	AEN	-0.314	-0.298	0.016	0	0	48	48
